# Identification of differentially expressed genes by means of outlier detection

**DOI:** 10.1186/s12859-018-2318-8

**Published:** 2018-09-10

**Authors:** Itziar Irigoien, Concepción Arenas

**Affiliations:** 1 Department of Computation Science and Artificial Intelligence, University of the Basque Country UPV/EHU, Donostia, Spain; 2 Department of Genetics, Microbiology and Statistics, University of Barcelona, Barcelona, Spain

**Keywords:** Differentially expressed gene, Multivariate statistics, Outlier, Quantile

## Abstract

**Background:**

An important issue in microarray data is to select, from thousands of genes, a small number of informative differentially expressed (DE) genes which may be key elements for a disease. If each gene is analyzed individually, there is a big number of hypotheses to test and a multiple comparison correction method must be used. Consequently, the resulting cut-off value may be too small. Moreover, an important issue is the selection’s replicability of the DE genes. We present a new method, called ORdensity, to obtain a reproducible selection of DE genes. It takes into account the relation between all genes and it is not a gene-by-gene approach, unlike the usually applied techniques to DE gene selection.

**Results:**

The proposed method returns three measures, related to the concepts of outlier and density of false positives in a neighbourhood, which allow us to identify the DE genes with high classification accuracy. To assess the performance of ORdensity, we used simulated microarray data and four real microarray cancer data sets. The results indicated that the method correctly detects the DE genes; it is competitive with other well accepted methods; the list of DE genes that it obtains is useful for the correct classification or diagnosis of new future samples and, in general, it is more stable than other procedures.

**Conclusions:**

ORdensity is a new method for identifying DE genes that avoids some of the shortcomings of the individual gene identification and it is stable when the original sample is changed by subsamples.

**Electronic supplementary material:**

The online version of this article (10.1186/s12859-018-2318-8) contains supplementary material, which is available to authorized users.

## Background

Analysis of gene expression data arising from microarray or RNA-Seq technologies is a very important task and a major advance in biomedical research. In this kind of experiments, the main goal is to identify a small number of informative genes whose patterns of expression differ according to the experimental conditions. These genes, selected from thousands, are differentially expressed, between two possible conditions, as control and treatment groups or between two groups of patients. This gene discovery is challenging, because there is a large number of genes, a relatively small number of samples and it is important to identify which genes, independently of the sample studied of the same disease, are selected as differentially expressed genes. The selection of relevant genes to differentiate these two conditions has two main objectives for researchers. On the one hand, to select a small number of genes so that the information given by these genes is not redundant, and the classification or diagnostic of new samples which lead to lower prediction error. On the other hand, a large number of selected genes are related to others that have the same function and that are highly correlated. It is clear that, in order to obtain a list of genes that allows a good diagnosis for future samples, a combination of these two objectives is desirable. It is also necessary to know the relation and function of the selected genes.

As, in general, the expression levels of genes are dependent on each other because genes are involved in complex regulatory pathways and networks [1], it seems convenient to consider the joint distribution of genes. However, methods to identify DE (differentially expressed) genes that are based on a gene-by-gene approach, ignoring the dependences between genes, are widely used. Maybe, the most popular is the *t*-test, but it has some restrictions [2, 3]. To solve the problem of unstable variances, the Significance Analysis of Microarrays (SAM) [2] was introduced. It works with a modified *t*-test introducing a factor to minimize the effect that small per-gene variances could make genes, with small differences between the expression conditions, statistically significant. An integrated solution for analyzing data from gene expression experiments is provided by limma [4, 5] an R package for Bioconductor [6]. limma fits a linear model for each gene and uses an empirical Bayes (eBayes) method for assessing differential expression. The empirical Bayes method (eBayes) [7] also uses moderated *t*-statistics, where instead of the global or single gene estimated variances, a weighted average of the global and single-gene variances is used. In the gene-by-gene approaches, as a large number of hypothesis tests are carried out, multiple testing procedures must be applied to assess the overall significance, controlling the family-wise error rate (FWER) or the false positive rate (FDR). The FWER is a very stringent criterion which measures the probability of at least one false positive in the set of significant genes, and most investigators accept a FWER of 5% [8]. A more liberal criterion is the FDR [9], which is the expected proportion of false positives among the significant genes. However, all the *p*-value adjustment methods lose sensitivity as they have a reduced chance of detecting true positives.

As different statistical selection methods may capture different statistical aspects of expression changes, they may give different lists of selected genes. Nevertheless, these inconsistent gene lists could be rather functionally consistent [10–12]. However, it is clear that one desirable property for a proper statistical method is that it detects true differentially expressed genes and has the ability to maintain a consistent list of differentially expressed genes within a single data set, that is, with samples based on subsets of the same data. In this direction, in [3] an empirical evaluation of consistency and accuracy for different methodologies was presented, concluding that for smaller sample sizes, moderated versions of the *t*-test can generally be recommended, while for large data sets, the method may involve a compromise between consistency and power.

A different approach was introduced in [13, 14]. In these works, the authors presented a statistic, called *OR*, which is useful to identify extreme observations or outliers in high-dimensional data sets. They presented the possibility to use the *OR* statistic as a tool to detect differentially expressed genes. The idea is that in expression studies, there are a large number of genes, and very few are expected to be important for the disease development. Thus, the important genes (which are DE) should show a different behaviour to those that are non-important. For this reason, the important genes could be considered as outliers in a background population of non-important genes.

One more fruitful endeavour than searching for lists of differentially expressed genes might be to search for the best way in which the two groups under study can be distinguished. Suppose that a new sample is considered, and we wish to decide which of the two groups it belongs to. The best set of differentially expressed genes will be the one that leads to the smallest probability of misclassifying this new sample, that is, the set of differentially expressed genes that leads to the smallest error rate of all future allocations of new samples. Thus, it is very interesting to analyze if a procedure obtains lists of differentially expressed genes useful for classification of future samples. It is therefore important to recognize the two objectives: to maximize separation between the groups of available samples, and to minimize the misclassification rate over all possible future allocations.

In this article, we present a new method which uses the *OR* statistics and two new measures which, together, are useful to obtain consistent lists of true differentially expressed genes, and which allows the correct classification of future samples. This novel approach, called ORdensity, takes into account the relation between all genes and it is not a gene-by-gene approach.

In the “Methods” section, we detail the basic ideas, the new concepts, the description of the new method, a small example to aid the comprehension of the new methodology, as well as the simulation studies and gene expression data from four public cancer studies, that were used to evaluate the behaviour of the procedure. In the “Results” and “Discussion” sections we show the usefulness of our approach. We close this paper with some conclusions.

## Methods

**Fig. 1:**
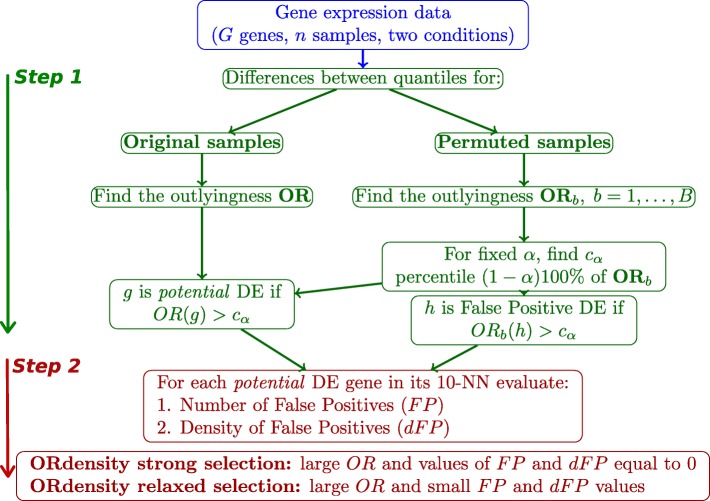
General outline of the proposed ORdensity approach. In green the first step of the method and in red the second step of the method

The new ORdensity procedure has two main steps: finding *potential* differentially expressed genes and identifying differentially expressed genes. Next, we detail these two steps of the method and Fig. 1 resumes the general outline of the approach. In order to better understand the procedure development, a small artificial example is also included.

Let *E*={**e**_1_,…,**e**_*G*_} be a set of expression level values for *G* genes such that each **e**_*g*_ is a vector **e**_*g*_=(**e**_*gX*_,**e**_*gY*_)^′^ giving the expression of the *g*-gene in two conditions X and Y (e.g., treatment/control or two patient groups). Then, $\mathbf {e}_{gX}=\left (e_{{gX}_{1}}, \ldots, e_{{gXn}_{X}}\right)^{\prime }$ and $\mathbf {e}_{gY}=\left (e_{{gY}_{1}}, \ldots, e_{{gYn}_{Y}}\right)^{\prime }$, *n*_*X*_+*n*_*Y*_=*n*, are vectors of values giving the expression of the *g*-gene in the *j*-sample under condition X and Y, respectively. Each **e**_*g*_ can then be considered a point in a continuous *n*-dimensional gene expression space *S*⊂R^*n*^. Let *X*_*g*_ and *Y*_*g*_ be the random variables representing the expression level of gene *g* in conditions X and Y, respectively (*g*=1,…,*G*).

**Fig. 2:**
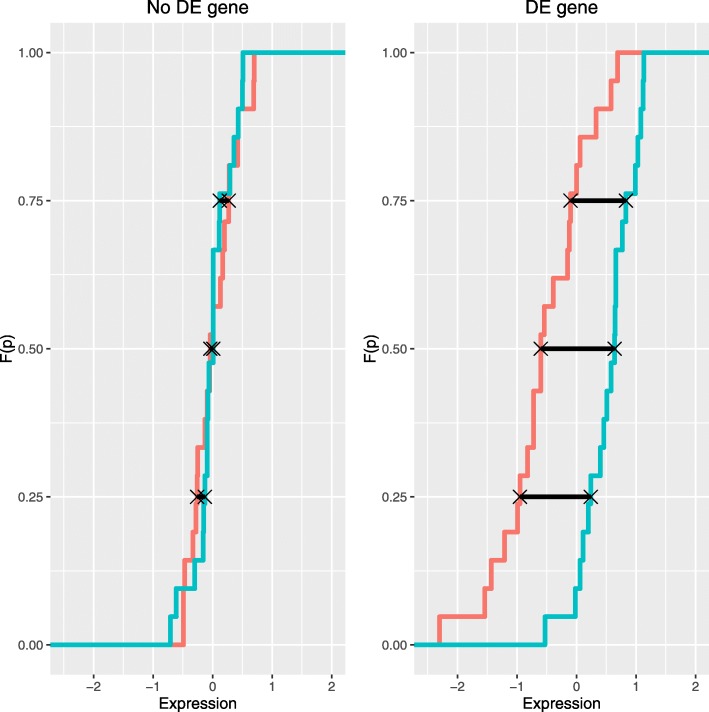
Visualization of $\hat {F}_{X_{g}}^{-1}(p)- \hat {F}_{Y_{g}}^{-1}(p)$ differences for *p*∈*C*_*p*_={0.25,0.5,0.75} for two genes. In the left side, a gene whose expressions in conditions X and Y are not differentially expressed (No DE gene); in the right side, a gene that is differentially expressed in conditions X and Y (DE gene)

The proposed approach focuses on the differences of quantiles between samples: $V_{gp} =F_{X_{g}}^{-1}(p)- F_{Y_{g}}^{-1}(p)$, *p*∈*C*_*p*_ where *C*_*p*_ is a set of probabilities. For instance, *C*_*p*_={0.25,0.5,0.75} is an adequate set for small sample sizes. A gene, *g*, whose expressions in conditions X and Y are considered not differentially expressed (see Fig. 2 left) would verify that $F_{X_{g}}^{-1}(p) = F_{Y_{g}}^{-1}(p)$, where *F* is the cumulative distribution function and *p*∈[0,1]. Otherwise, gene *g* is differentially expressed (DE) or it is important (see Fig. 2 right). Broadly speaking, matrix **V**=(*v*_*gp*_) with *v*_*gp*_=$ \hat {F}_{X_{g}}^{-1}(p)- \hat {F}_{Y_{g}}^{-1}(p)$, for *g*=1,…,*G* and *p*∈*C*_*p*_ must contain small values corresponding to the major number of no DE genes. However, the most differentially expressed genes should show a different behaviour, and for this reason they can be considered as outliers in **V**. Thus, our approach attempts, in two main steps, to find outliers in **V** which can be identified as differentially expressed genes.

### First step: finding *potential* differentially expressed genes

Let **V**=(*v*_*gp*_) the *G*×*P* matrix with $v_{gp} =\hat {F}_{X_{g}}^{-1}(p)- \hat {F}_{Y_{g}}^{-1}(p)$, *p*∈*C*_*p*_ where *C*_*p*_ is a set of probabilities (*P*=*#C*_*p*_). As DE genes are expected to be outliers based on the $\mathbf {v}_{g} = \left (v_{gp}\right)'_{p \in C_{p}}$ values, the procedure computes the robust index *OR* of outlyingness [13, 14] as follows. Define *d*_*gh*_ the Euclidean distance between **v**_*g*_ and **v**_*h*_. For a fixed gene *g*, the *OR* statistic is given by:

$$OR(g)=\frac{{Median}_{h = 1, \ldots,G}\left\{d^{2}_{gh}\right\}}{1/2 {Median}_{h, \, j =1, \ldots, G}\left\{d^{2}_{hj}\right\}} $$

In this ratio, the numerator gives the median value of all (squared) distances of the gene of interest with respect to all the other genes; the denominator gives (half) the median of all (squared) distances among all genes. As a consequence, given a set of *G* genes, *OR* gives a ranking of genes, so that genes with a large value of *OR* will be genes that are further away from the set of all genes and therefore they are possible outliers (i.e., important genes). In this way, the original *G*×*n* data matrix is firstly transformed in a *G*×*P* matrix with the *v*_*gp*_ differences between *C*_*p*_-quantiles and, secondly, in a *G* dimensional vector with the *OR* values **OR**=(*OR*(*g*_1_),…,*OR*(*g*_*G*_))^′^. As the distribution of **OR** is unknown, the procedure considers permuted samples in order to generate values associated with genes which are not differentially expressed. Thus, the expression values of each gene are permuted *B* times, that is, each sample has its corresponding label and actually the permutation is carried out on the labels. Once the labels of the samples are reassigned by permutation (B times), the expressions of the genes are classified according to those two classes. Then, the procedure computes matrix ${\mathbf {V}}_{b}^{*}$ and vector **OR**_*b*_=(*OR*_*b*_(*g*_1_),…,*OR*_*b*_(*g*_*G*_))^′^ for each permutation *b*, (*b*=1,…,*B*). Given a fixed *α*∈(0,1), it calculates the percentile (1−*α*) 100*%* of all the elements of the matrix with the permuted samples **OR**_*b*_. Let us denote this value by *c*_*α*_. In this way, the method excludes those genes, *g*, with *OR*(*g*)≤*c*_*α*_. Then, DE genes must be in the subset of genes *S*_*α*_={*g* | *OR*(*g*)>*c*_*α*_}. We call them *potential* genes.

### Second step: identifying differentially expressed genes

By construction, there are *α*×*G*×*B* cases where *OR*_*b*_(*h*)>*c*_*α*_ among the permuted samples and we call *R*_*α*_={*h* | *OR*_*b*_(*h*)>*c*_*α*_, *b*=1,…*B*} the set of those cases. That is, in *R*_*α*_ there are all permuted cases *h* with *OR*_*b*_(*h*)>*c*_*α*_ for some *b*. These cases are related to genes that are not DE but have large *OR* values. The corresponding values of ${\mathbf {v}}_{b, h}^{*}$, reflect the behaviour of these genes in relation to the differences between quantiles. Hence, we have, on the one hand, $\left (\mathbf {v}_{g}'\right)_{g \in S_{\alpha }}$ differences for *potential* differentially expressed genes and on the other hand, we know that the differences $\left (\mathbf {v}_{b,\,h}^{*}\right)_{h \in R_{\alpha }}$ represent the behaviour of cases that are false positives with a misleadingly large value of *OR*. Therefore, the analysis of the differences and similarities between **v**_*g*_ and $\mathbf {v}_{b,\,h}^{*}$ will provide a way to discriminate the truly DE genes among the set *S*_*α*_ of *potential* DE genes.

To this aim, consider matrix $\left (\mathbf {v}_{b,\,h}^{*}\right)_{h \in R_{\alpha }}$ randomly divided into *k*-folds. Then, consider the union of set *S*_*α*_ with the *i*th fold *R*_*α*,*i*_={*h*∈*R*_*α*_| *i*th fold}, that is, *U*_*i*_=*S*_*α*_∪*R*_*α*,*i*_. In order to understand what happens in *U*_*i*_, consider the following small artificial example.

**Fig. 3:**
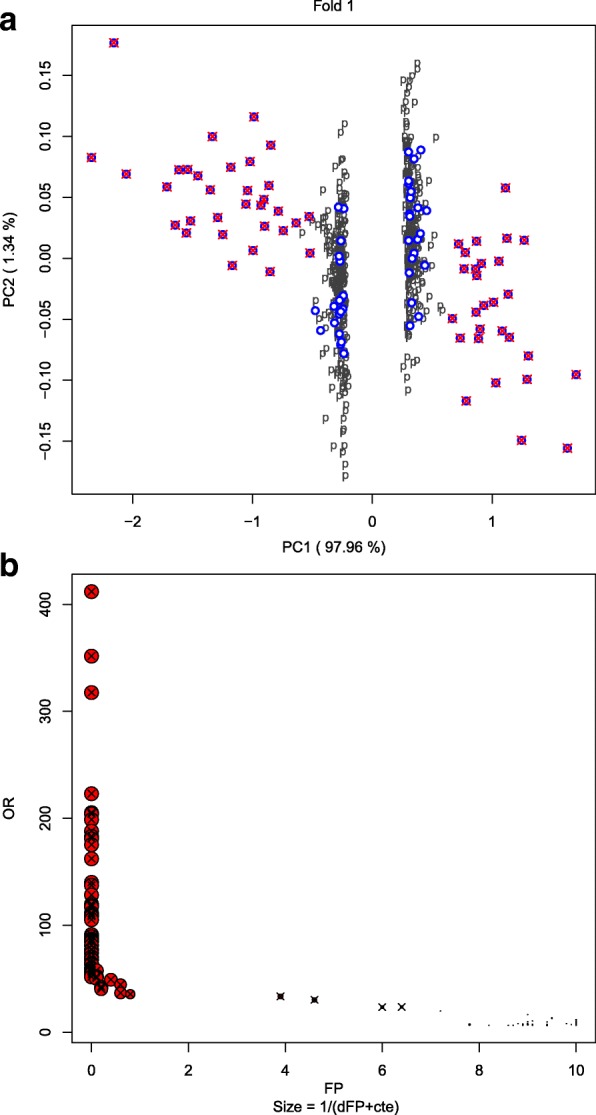
Illustrative example. **a** First two principal components of data $\left [(\mathbf {v}_{g})_{g \in S_{\alpha }} \, | (\mathbf {v}_{b,h}^{*})_{h \in R_{\alpha \,, 1}}\right ]'$ corresponding to fold 1 (99.3% of explained variability), and there are represented: the *potential* genes (genes in *S*_0.05_) by circles; the false positives genes (genes in *R*_*α*,*i*_) by “p"s, and the differentially expressed genes (genes generated as truly DE genes) by crosses. **b** Representation of the *potential* genes based on *OR* (vertical axis), *FP* (horizontal axis) and *dFP* (size of the circle is inversely proportional to its value). Truly DE genes are marked with a cross; in red and blue, genes belonging to cluster 1 and cluster 2, respectively

*Small artificial example:* We simulated [15] 1000 genes under two conditions X and Y with 30 samples for each condition and 60 of these genes were generated as differentially expressed. We considered *C*_*p*_={0.25,0.5,0.75} to build the (1000×3)-matrix **V** of differences between the quartiles and these differences were weighted with {0.25,0.5,0.25}, respectively. The procedure obtained the matrix **V** of differences between the weighted quartiles and it calculates the *OR* statistic for the 1000 original genes, **OR**=(*OR*(*g*_1_),…,*OR*(*g*_1000_))^′^. Next, for *B*=100 permuted samples of *X* and *Y*, we computed matrix ${\mathbf {V}}_{b}^{*}$ of differences between the weighted quartiles and we computed their *OR* values **OR**_*b*_=(*OR*_*b*_(*g*_1_),…,*OR*_*b*_(*g*_1000_)), *b*=1,…,*B*. For a fixed *α*=0.05, the percentile (1−*α*) 100*%* of **OR**_*b*_ on the *B*=100 permuted samples was *c*_0.05_=6.27 and the set *S*_0.05_={*g* | *OR*(*g*)>*c*_0.05_}={*g* | *OR*(*g*)>6.27} contained 100 genes, which were *potential* DE genes. Furthermore, between the 1000×100 permuted observations, 5000 of them had an *OR* value above the threshold *c*_0.05_=6.27. Next, we considered a 10-fold partition. For fold 1, the Fig. 3a shows the results of the two first principal components analysis on the matrix $\left [\left (\mathbf {v}_{g}\right)_{g \in S_{\alpha }} \, | \left (\mathbf {v}^{*}_{b,\,h}\right)_{h \in R_{\alpha, i}}\right ]'$, with 99.3% of explained variability. As can be observed, a *potential* gene *g* that is not really differentially expressed was closely surrounded by cases from *R*_*α*,*i*_, that is, by false positive permuted cases. On the contrary, a gene *g* that is really differentially expressed was not surrounded by cases from *R*_*α*,*i*_. That holds for every fold and clearly shows the intuitive idea behind the proposed methodology.

Following with the method, let us call (*f*_*i*_,*f*_*i*0_) the proportion of *potential* genes and permuted cases in set *U*_*i*_ (*i*=1,…,*k*). As we have observed in the small artificial example, if a *potential* gene is genuinely DE, its behavior should be different from those cases in *R*_*α*,*i*_ and the other way round; if a *potential* gene presents a similar behavior to those cases in *R*_*α*,*i*_, then it should be considered as not truly DE. So, for each gene *g* in *S*_*α*_, its 10-Nearest-Neighbourhood (*NN*_*i*_(*g*)) in *U*_*i*_ is considered. We calculate two indicators in this neighbourhood: the number of cases from *R*_*α*,*i*_, *FP*_*i*_(*g*)=*#*{*j*∈*NN*_*i*_(*g*)|*j*∈*R*_*α*,*i*_} (number of false positive permuted cases) and the density ${dFP}_{i}(g) = {FP}_{i}(g)/\max _{j \in {NN}_{i}(g)}\left \{d_{gj}^{2}\right \}$. The denominator $\max _{j \in {NN}_{i}(g)}\left \{d_{gj}^{2}\right \}$ will rarely be 0 since it would involve 10 tied nearest distances for g in *NN*_*i*_(*g*), but if it is equal to 0 then *dFP*_*i*_(*g*)=0 if *FP*_*i*_(*g*)=0 and *dFP*_*i*_(*g*)=NaN if *FP*_*i*_(*g*)>0.

Gathering the *k* folds, we obtain for each *g*∈*S*_*α*_ the average number of false positive permuted cases (*FP*) and the average density (*dFP*) of *FP* in the neighbourhood: $FP(g)=1/k\sum _{i}{FP}_{i}(g)$ and $dFP(g)=1/k\sum _{i}{dFP}_{i}(g)$.

Thus, to discriminate DE genes among those in set *S*_*α*_, we have to look for those *g* with low value of *FP*(*g*) and *dFP*(*g*) along with high values of *OR*(*g*).

Under this criterion, two types of differentially expressed gene selection can be made:

- **ORdensity strong selection:** take as differentially expressed genes those with a large *OR* value and with *FP* and *dFP* equal to 0.
- **ORdensity relaxed selection:** take as differentially expressed genes those with a large *OR* value and with small *FP* and *dFP* values. The average proportion of *potential* genes and the permuted cases among the *k* folds ($f=1/k\sum f_{i}$ and $f_{0}=1/k\sum f_{i0}$ respectively) are a good reference to look for small values of *FP*.

Furthermore, beyond the inspection of individual genes, we tackled the selection of DE genes by clustering the genes in *S*_*α*_ based on the input variables *OR*, *FP* and *dFP*. This can be useful as it offers different patterns of genes based on their importance.

*Small artificial example (cont.):* Using the Partition Around Medoids (PAM) clustering method [16] on variables *OR*,*FP* and *dFP*, and selecting two clusters as indicated in the silhouette analysis [17], the variables presented the following basic characteristics (Table 1) and the two clusters are represented in Fig. 3b. It is worth mentioning that the average distribution of *potential* genes and permuted cases in sets *U*_*i*_ was $\left (\sum _{i}f_{i}/10, \sum _{i}f_{i0}/10) = (0.17, 0.83\right)$. It means that if the distribution were random, an average of 8.3 permuted cases would be expected in the 10-NN. Clearly, in cluster 1 the *FP* values are below this value. Finally, we checked the distribution of real DE genes across the two clusters, and the 60 genes in Cluster 1 are exactly the 60 real DE genes.

**Table 1:** Illustrative example

	Cluster 1 (*n*_1_=60)			Cluster 2 (*n*_2_=40)		
	*OR*	*FP*	*dFP*	*OR*	*FP*	*dFP*
Min	23.5	0.0	0.00	6.3	7.1	8.13
*Q*_1_	53.8	0.0	0.00	6.7	9.0	24.78
*Q*_2_	79.3	0.0	0.00	7.3	9.1	29.13
Mean	104.5	0.4	0.25	8.5	9.2	28.51
*Q*_3_	122.1	0.0	0.00	9.6	9.7	32.78
Max	412.0	6.7	5.22	19.8	10.0	43.21

Basic description for the *OR*,*FP* and *dFP* values in the two clusters obtained using PAM and silhouette procedures

***Note 1:*** When the variability of genes among different types of samples is different, it is advisable to scale the differences between quantiles, for instance, $v_{gp} =\left (\hat {F}_{X_{g}}^{-1}(p)- \hat {F}_{Y_{g}}^{-1}(p)\right)/\max \{RI(X_{g}), RI(Y_{g})\}$, with *RI*(*X*_*g*_) and *RI*(*Y*_*g*_) the interquartile ranges in samples X and Y, respectively.

***Note 2:*** As the method considers the differences between quantiles, it may be interesting to give greater importance to some of them, such as the median. Simultaneously, robustness can be obtained avoiding the possible fluctuation of the estimations of the percentiles. Therefore, different weights to the quantiles can be introduced in the procedure.

***Note 3:*** We have considered for each gene *g* in *S*_*α*_ its 10-Nearest-Neighbourhood. This is a parameter of the method that could be set in different ways, and it is considered to obtain better estimations of the proportion of potential genes and permuted cases in set *U*_*i*_. In this sense, a small number of neighbors as 5 does not seem adequate since the percentage of false positive permuted cases that could be detected would be very unstable. On the other hand, note that as the method takes into account the mean value of the false positive density, the possible effect of the number of chosen neighbors is minimized.

### Experimental setup

To evaluate the usefulness of the ORdensity procedure, we simulated multiple gene expression data sets using different parameter settings. Furthermore, we applied the procedure to four real cancer data sets. All computations were performed using the R language and Environment for Statistical Computing (R) 3.3.1 [18, 19] in combination with Bioconductor 3.3 [6]. As the proposed method may depend on the quantiles estimation of each gene expression for different conditions, different sample size situations including the case of small samples were considered in the simulated studies. Moreover, for both simulated and actual data, the method was compared with other well-recognized methods in order to assess whether it could compete with them.

#### Simulation study 1

We assumed a total of 1000 genes, among which 50, 100 or 200 were differentially expressed (DE) genes. On the one hand, the expression levels of all no DE genes were generated by $N\left (0, \sigma _{g}^{2}\right)$ and $N\left (0, \gamma _{g}^{2}\right)$ distributions in conditions X and Y, respectively. On the other hand, the DE genes were generated using the $N\left (0, \alpha _{g}^{2}\right)$ and $N\left (\mu _{g}, \beta _{g}^{2}\right)$ distributions for conditions X and Y, respectively, with |*μ*_*g*_|=*Δ*_*g*_ max{*α*_*g*_,*β*_*g*_}. Parameter *Δ*_*g*_ sets the importance of gene *g*, being gene *g* more important as long as *Δ*_*g*_ is bigger. Within this general setting, three different scenarios were considered:

**Scenario 1:** All genes had equal variability (*σ*_*g*_ = *γ*_*g*_ = 1,*α*_*g*_ = *β*_*g*_=1), and all DE genes are equally important under this scenario, i.e., *Δ*_*g*_=*Δ*, with *Δ* in {1.5,2,3}.

**Scenario 2:** All genes had not necessarily equal variability (*σ*_*g*_≠*γ*_*g*_,*α*_*g*_≠*β*_*g*_). These variabilities were randomly selected among {1,1.2,1.5,2}. All DE genes are equally important, i.e., *Δ*_*g*_=*Δ* for all *g*, with *Δ* in {1.5,2,3}.

**Scenario 3:** All genes had not necessarily equal variability (*σ*_*g*_≠*γ*_*g*_,*α*_*g*_≠*β*_*g*_) neither the importance of DE genes is the same (*Δ*_*g*_≠*Δ*_*h*_, *g*≠*h*). Variability parameters were randomly selected in {1,1.2,1.5,2} as in the previous scenario and *Δ*_*g*_ values were randomly selected among {1.5,2,3}.

To better understand the performance of our approach, we simulated the data for the three scenarios assuming equal sample sizes for X and Y (*n*_*X*_=*n*_*Y*_=30) and different sample sizes (*n*_*X*_=30, *n*_*Y*_=10). For the most general situation, scenario 3, we also considered the case *n*_*X*_=*n*_*Y*_=10 in order to evaluate the procedure for small sample sizes.

Using the area under the ROC curve, for the three scenairos and for *n*_*X*_=*n*_*Y*_=30, *n*_*X*_=30, *n*_*Y*_=10 and *n*_*X*_=*n*_*Y*_=10, the ORdensity results were compared with those obtained using other well-known methods in this field, such as Significant Analysis of Microarrays [2] and Linear models with Empirical Bayes statistic (limma [4, 5]). Furthermore, for each situation 100 replicates were performed.

The Significant Analysis of Microarrays (SAM) method is a modification of the t-statistic and it was introduced to avoid the effect of the small per-gene variances that can make small fold-changes statistically significant. This modification adds a value which is calculated from the distribution of gene-specific standard errors. SAM was applied using the package samr for Bioconductor [6] in R language and Environment for Statistical Computing [18, 19].

The Empirical Bayes statistic (eBayes) is equivalent to shrinking the estimated sample variances towards a pooled estimate, resulting in far more stable inference when the number of arrays is small. The linear model with empirical Bayes statistic (called limma in the following) was applied using the limma package for Bioconductor [6] in R language and Environment for Statistical Computing [18, 19].

#### Simulation study 2

The simulation was set with blocks of DE genes [20] which are correlated within each block. The data was simulated from a multivariate normal distribution, with all genes having variance 1 and correlation 0.9 between genes within each block. It means that the variance-covariance matrix was a block-diagonal matrix such as:

$$\Sigma = \left(\begin{array}{cccc} \mathbf{\sigma}_{block} & \mathbf{0} & \ldots & \mathbf{0}\\ \mathbf{0} & \mathbf{\sigma}_{block} & \ldots & \mathbf{0}\\ \vdots & \vdots & \vdots & \vdots\\ \mathbf{0} & \mathbf{0} & \ldots & \mathbf{\sigma}_{block}\\ \end{array} \right)$$

where **σ**_*block*_=(*σ*_*gh*_)_*gh*_ with *σ*_*gg*_=1 and *σ*_*gh*_=0.9*g*≠*h*.

We considered 1, 2 or 3 blocks, and each block with 5, 20 or 100 DE genes. Following [20], the difference between mean values of conditions X and Y was set depending on the number of blocks:

**One block:** *μ*_*A*_=−1.65, *μ*_*B*_=1.65

**Two blocks:** **μ**_*A*_=(−1.18,−1.18)^′^, *μ*_*B*_=(1.18,1.18)^′^

**Three blocks:** **μ**_*A*_=(−0.98,−0.98,−0.98)^′^, *μ*_*B*_=(0.98,0.98,0.98)^′^

Once the DE genes were generated, 4000 variables representing no DE genes were added: 2000 following a *N*(0,1) distribution and 2000 following a $\mathcal {U}(-1, 1)$ distribution.

For the different number of blocks, we considered equal sample sizes for X and Y (*n*_*X*_=*n*_*Y*_=30) and different sample sizes (*n*_*X*_=30, *n*_*Y*_=10). For each situation 100 replicates were done and using the area under the ROC curve, the OR results were compared with those obtained by limma and SAM.

In all the above simulations, in order to obtain comparable results throughout the 100 runs, we always considered 3 clusters determined by PAM [16] clustering procedure. Obviously, to be absolutely accurate it would have been necessary to determine the number of clusters in each dataset.

#### Actual data sets: lymphoma, Golub, colon and prostate cancer

We considered four publicly available cancer data sets: a well-known lymphoma data set which is in the R package spls, and post-processed Golub, colon and prostate data sets that were downloaded from [21]. With these data sets, we compared the ORdensity results with SAM and limma.

The standard rule used for selecting a gene as DE with limma was to present an adjusted *p*-value smaller than 0.05. To compute the adjusted *p*-values for gene ranking we used a very stringent method (Bonferroni), and a more liberal procedure (BH, [9]). For SAM, the considered rule was to present a q-value [22] equal to 0.

For the ORdensity procedure, the Partition Around Medoids (PAM) [16] clustering and the silhouette analysis [17] were performed in order to establish the number of clusters and both the strong and relaxed selection were considered.

We evaluated the obtained results considering three different perspectives: the agreement between the three methods of the selected gene lists; the ability to maintain consistent lists with samples based on subsets of 80% of the original data selected at random, and the leave-one-out cross-validation correct classification rate for future classifications obtained when a Weighted Distance Based Discriminant analysis (WDB-discriminant) using Euclidean distance was performed [23]. Weighted Distance Based Discriminant (WDB-discriminant) is an improvement of the Distance Base rule [24, 25] which takes into account the statistical depth of the units. The WDB-discriminant was applied using the WeDiBaDis package available at https://github.com/ItziarI/WeDiBaDis.

#### Lymphoma cancer data set:

This data set [26] contains the gene expression of 1095 genes measured on 42 adults with large B-cell lymphoma (DLBCL), which can present two different molecular forms denoted by DLBCL1 and DLBCL2, respectively. Half of the samples presented the form DLBCL1 and the other half the form DLBCL2.

#### Golub data set:

The data set [27] contains the gene expression of 7129 genes. There are 72 samples with two different types of leukaemia, 47 acute lymphoblastic leukaemia (ALL) and 25 with acute myeloblastic leukaemia (AML).

#### Colon cancer data set:

This colon cancer data study [28] consists of 6000 genes measured on 62 patients, 40 of them diagnosed with colon cancer and 22 of them are healthy.

#### Prostate cancer data set:

This study [29] considered 12,626 genes and 102 samples, 50 of which were non-tumour prostate samples and 52 of which were prostate tumours.

In all the experiments, simulated or actual data sets, we considered the differences between the three quartiles, that is, *C*_*p*_={0.25,0.5,0.75}. Moreover, we scaled these differences and weighted them by {1/4,1/2,1/4} respectively, making the most important difference the one between the medians (See Note 1 and 2, in the “Methods” section). In the case of the Golub data set, these differences were not scaled because for some of the genes the interquartile range was null.

## Results

Next, we present the results obtained with the simulated data, as well as the actual cancer data sets.

With the simulated microarray data, we evaluated the behavior of the method in relation to the correct selection of DE genes since, in this case, we know which genes are actually differentially expressed. We evaluated the general behavior of the procedure in relation with the proportion of False Positive among the selected genes as DE and using the area under the ROC curve value, we compared the ORdensity results with those obtained using the alternative approaches, SAM and limma. Furthermore, we present a detail evaluation of both, the first and the second step of the method.

With the actual microarray data, we evaluated and compared our procedure with the alternative approaches SAM and limma, measuring the ability of the method in preserving predictive accuracy classifying the samples, and the stability of the lists of selected genes when a fraction of the samples (20%) was eliminated at random.

### Simulation study 1

#### General behavior

As a general summary, the results indicated that the three variables that the method builds, *OR*, *FP* and *dFP*, are good discriminative variables, separating correctly the DE genes from the not DE genes. On the one hand, the number of DE genes not detected by the method was very small and always related with the less DE genes (see “Detail evaluation of step 1” subsection). On the other hand, with the strong selection no False Positives were detected. With the more relaxed selection and considering the partition in three clusters for all the runs, the results indicated that, in all situations, the method only considers as DE genes those from clusters 1 and 2. The False Positives were mostly in cluster 2 and the worst results were obtained with 50 simulated genes and for small sample sizes (equal to 10). However, for the majority of the situations the average number of False Positives was 0 in cluster 1 and very small in cluster 2. It is worth to mention that partition in 3 clusters is not necessarily the best partition that could be obtained in each of the 100 runs. As a consequence, the variability of the percentage of False Positives in cluster 2 was very high for some cases. That is, for some of the runs, cluster 2 was formed by DE genes but not for other runs, for which a partition with different number of clusters would have probably been more appropriate (see Detail evaluation of step 2 subsection).

#### Detail evaluation of step 1

Regarding this step, it is necessary to evaluate the number of simulated DE genes that the method did not consider as *potential* genes and therefore were missed. The results indicated that as the number of simulated DE genes increases, it is easy to not include at least one DE gene in the set of *potential* DE genes. Moreover, when the sample size for one or the two conditions was small (equal to 10), the sensibility to include among the *potential* all the simulated DE genes decreases. Nevertheless, the proportion of DE genes considered as *potential* DE genes was always very large, and DE genes not considered as *potential* genes were very few and always associated with *Δ*=1.5.

**Fig. 4:**
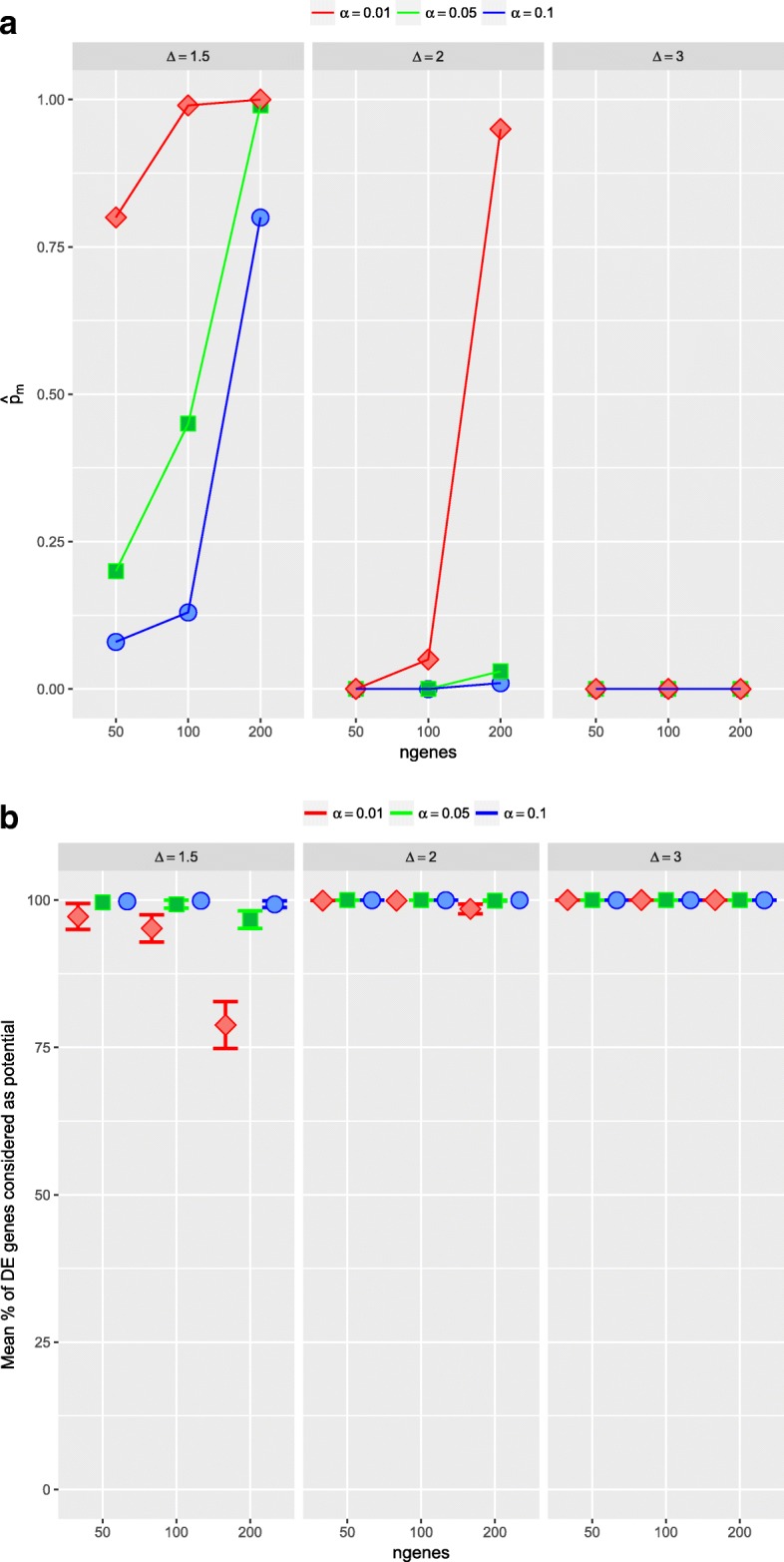
Simulation study 1, scenario 1 with *n*_*X*_=*n*_*Y*_=30 and 100 runs. Evaluation of the first step of the ORdensity method using different values of *α*. Top: in *x* axis number of DE genes; in *y* axis estimated probability, $\hat {p}_{m}$, of no considering as *potential* DE gene at least one gene that it really is. Bottom: in *x* axis number of DE genes; in *y* axis mean proportion of DE genes that the procedure considered as *potential* DE genes

With more detail, on the simulated data sets with equal sample sizes for the two conditions (*n*_*X*_=*n*_*Y*_=30), and in scenarios 1 and 2, when all the simulated DE genes were related to the same value of the parameter *Δ*_*g*_ (*Δ*_*g*_=*Δ* for all DE genes), we can observe that as the number of simulated DE genes increases, it is easy to not include at least one DE gene in the set of *potential* DE genes (Table 2, Fig. 4 and Additional file 1: Table S13, Figure S8). However, it is clear that, in any case, the proportion of DE genes considered as *potential* DE genes is very large, being always higher than 95% and only in two cases presents a lower value (78.80% and 85.24%, respectively). Moreover, these worst results were associated with the lowest value of *Δ*, specifically *Δ*=1.5. In scenario 3, where the importance of DE genes is different and set by their values *Δ*_*g*_ selected at random in {1.5,2,3}, it is interesting to analyze what is the importance of the genes that are not considered as *potential* in set *S*_*α*_. For instance, in the case of 50 DE genes, on average 99.32% of them are included in *S*_0.01_. Moreover, the DE genes not detected as *potential* had *Δ*_*g*_=1.5, the lowest value. Thus, all the DE genes not considered as *potential* in *S*_0.01_ are between the less DE. Similar results are observed in the rest of the Table (Additional file 1: Table S14, and Figure S9).

**Table 2:** Simulation study 1, scenario 1 with *n*_*X*_=*n*_*Y*_=30 and 100 runs

			*Δ*=1.5	
Nb. of			*α*	
DE genes		0.1	0.05	0.01
50	$\hat {p}_{m}$	0.08	0.20	0.80
	%	99.84 (0.54)	99.56 (0.92)	97.20 (2.2)
100	$\hat {p}_{m}$	0.13	0.45	0.99
	%	99.87 (0.34)	99.34 (0.66)	95.20 (2.3)
200	$\hat {p}_{m}$	0.80	0.99	1.00
	%	99.27 (0.56)	96.70 (1.5)	78.80 (4.0)
			*Δ*=2	
Nb. of			*α*	
DE genes		0.1	0.05	0.01
50	$\hat {p}_{m}$	0.00	0.00	0.00
	%	100	100	100
100	$\hat {p}_{m}$	0.00	0.00	0.05
	%	100	100	99.95 (0.22)
200	$\hat {p}_{m}$	0.01	0.03	0.95
	%	100	99.98 (0.09)	98.50 (0.83)
			*Δ*=3	
Nb. of			*α*	
DE genes		0.1	0.05	0.01
50	$\hat {p}_{m}$	0.00	0.00	0.00
	%	100	100	100
100	$\hat {p}_{m}$	0.00	0.00	0.00
	%	100	100	100
200	$\hat {p}_{m}$	0.00	0.00	0.00
	%	100	100	100

Evaluation of the first step of the ORdensity method using different values of *α*. The Table shows the estimated probability, $\hat {p}_{m}$, of no considering as *potential* DE gene at least one gene that it really is, and the mean proportion of DE genes (row named “%”) that the procedure considered as *potential* DE genes. Corresponding standard deviations are in brackets

**Fig. 5:**
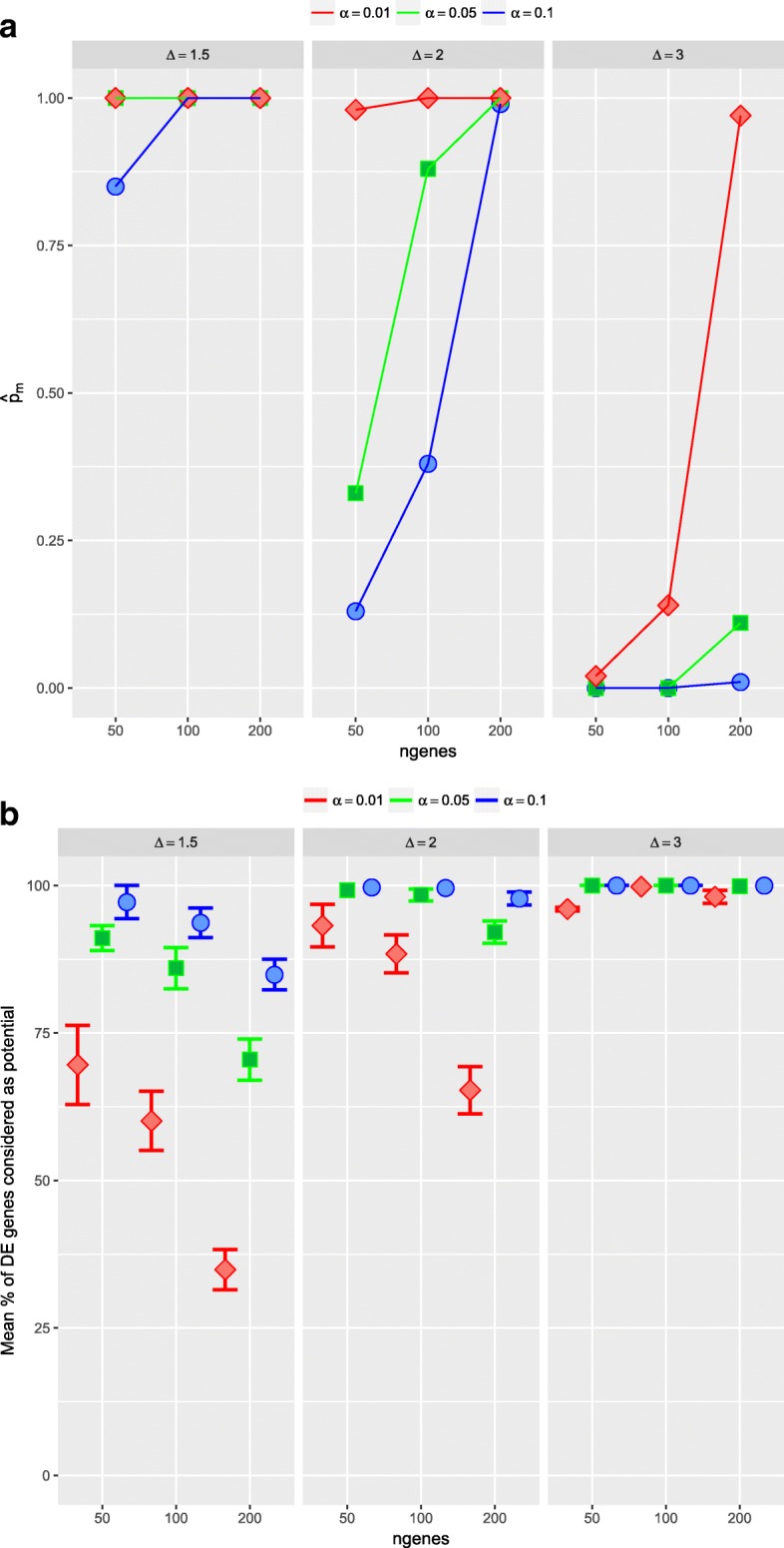
Simulation study 1, scenario 1 with *n*_*X*_=30, *n*_*Y*_=10 and 100 runs. Evaluation of the first step of the ORdensity method using different values of *α*. Top: in *x* axis number of DE genes; in *y* axis estimated probability, $\hat {p}_{m}$, of no considering as *potential* DE gene at least one gene that it really is. Bottom: in *x* axis number of DE genes; in *y* axis mean proportion of DE genes that the procedure considered as *potential* DE genes

In the case where the sample sizes for the two conditions were different (*n*_*X*_=30,*n*_*Y*_=10) and for the three scenarios, as we can observe, the results were very similar. As the sample size in one condition is smaller, the sensibility to include among the *potential* all the DE genes decreases, that is, the probability of not considering as *potential* DE gene at least one DE gene really increases (Table 3, Fig. 5 and Additional file 1: Tables S15 and S16, Figures S10 and S11).

**Table 3:** Simulation study 1, scenario 1 with *n*_*X*_=30, *n*_*Y*_=10 and 100 runs

			*Δ*=1.5	
Nb. of			*α*	
DE genes		0.1	0.05	0.01
50	$\hat {p}_{m}$	0.85	1	1
	%	97.2 (2.8)	91.1 (2.1)	69.6 (6.7)
100	$\hat {p}_{m}$	1	1	1
	%	93.7 (2.5)	86.0 (3.5)	60.1 (5.0)
200	$\hat {p}_{m}$	1	1	1
	%	84.9 (2.6)	70.5 (3.5)	34.9 (3.4)
			*Δ*=2	
Nb. of			*α*	
DE genes		0.1	0.05	0.01
50	$\hat {p}_{m}$	0.13	0.33	0.98
	%	99.7 (0.7)	99.2 (1.2)	93.2 (3.6)
100	$\hat {p}_{m}$	0.38	0.88	1
	%	99.6 (0.6)	98.4 (1.0)	88.4 (3.2)
200	$\hat {p}_{m}$	0.99	1	1
	%	97.8 (1.1)	92.1 (1.9)	65.3 (4.0)
			*Δ*=3	
Nb. of			*α*	
DE genes		0.1	0.05	0.01
50	$\hat {p}_{m}$	0.00	0.00	0.02
	%	100	100	96.0 (0.3)
100	$\hat {p}_{m}$	0.00	0.00	0.14
	%	100	100	99.8 (0.5)
200	$\hat {p}_{m}$	0.01	0.11	0.97
	%	100.0 (0.1)	99.9 (0.2)	98.1 (1.1)

Evaluation of the first step of the ORdensity method using different values of *α*. The Table shows the estimated probability, $\hat {p}_{m}$, of no considering as a *potential* DE gene at least one gene that it really is, and the mean proportion of DE genes (row named “%”) that the procedure considered as *potential* DE genes. Corresponding standard deviations are in brackets

For scenario 3, when both sample sizes are small (*n*_*X*_=*n*_*Y*_=10) similar results were obtained. Nevertheless, the mean proportion of DE genes that were included in the *potential* was notably lower. Again, the missed DE genes were mostly related to *Δ*_*g*_ = 1.5 (Additional file 1: Table S17 and Figure S12).

#### Detail evaluation of step 2

In the second step of the procedure, the interest lies in evaluating the number of False Positive genes selected by the method. As a general summary, with the strong selection no False Positive were selected. When the method used the more relaxed selection it retained as DE, in all cases, genes in clusters 1 and 2. The False Positive genes were mostly in cluster 2 and the worst results were obtained with 50 simulated genes and for small sample sizes (equal to 10). As mentioned before, partition in 3 clusters, throughout all runs, may lead to a high variability in cluster 2.

**Fig. 6:**
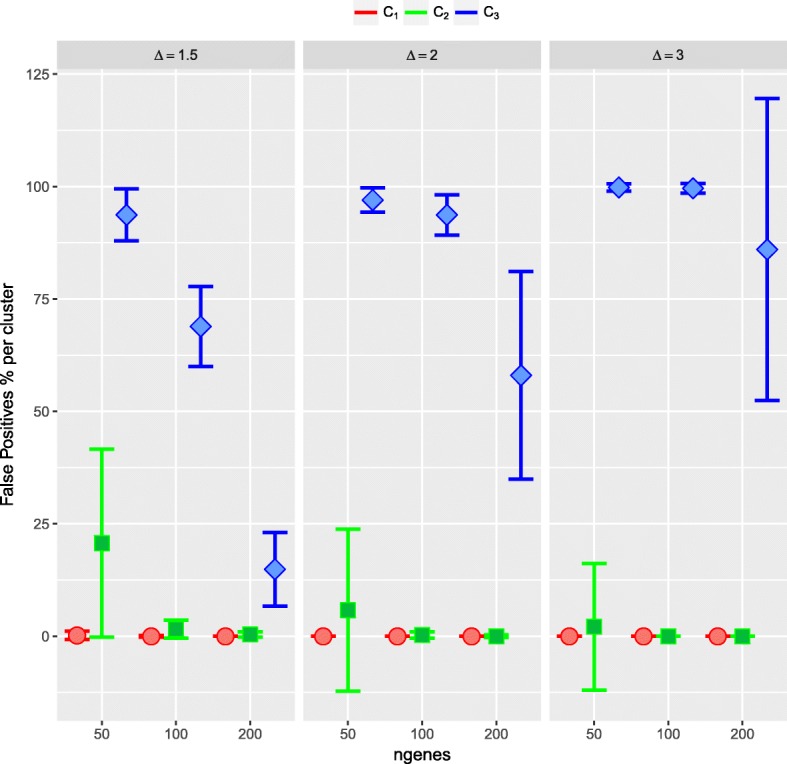
Simulation study 1, scenario 1 with *n*_*X*_=*n*_*Y*_=30 and 100 runs. Evaluation of the second step of the ORdensity method with *α*=0.05. In *x* axis number of DE genes; in *y* axis the mean of False Positives genes per cluster in % ($\overline {FPC}$). In red cluster 1 (C_1_), in green cluster 2 (C_2_) and in blue cluster 3 (C_3_)

Focusing on the details, it can be seen that in scenario 1, *n*_*X*_=*n*_*Y*_=30, *Δ*=1.5, and for any number of simulated DE genes, by chance an average of 8.5 False Positive permuted cases would be expected in the 10-NN. For 50 simulated DE genes, clearly, in clusters 1 and 2 the mean FP value is below 8.3. Then, the method considered genes in clusters 1 and 2 as DE, with on average less than one False Positive gene in cluster 1 (0.2% in cluster 1) and an average of 4.5 False Positive genes in cluster 2 (20.7% in cluster 2). When the number of simulated DE genes increases, a small number of False Positive genes were obtained. For 100 simulated DE genes, again the method considered those in clusters 1 and 2 as DE genes, with 0 False Positive in cluster 1 and on average less than one False Positive gene in cluster 2 (1.6%). Similar results were obtained for 200 simulated DE genes. For greater values of *Δ*, and for any number of simulated DE genes, the average number of False Positives per cluster is 0 or very small, being in the worst case equal to 1.6 (Table 4, Fig. 6). With equal sample sizes for the two conditions (*n*_*X*_=*n*_*Y*_=30), in scenarios 2 and 3, again, small values of the average number of False Positives per cluster were obtained, being 0 or less than one in the majority of the situations. In the worst situation related to 50 simulated DE genes, the average number of False Positives per cluster was 1.47.

**Table 4:** Simulation study 1, scenario 1 with *n*_*X*_=*n*_*Y*_=30 and 100 runs

*Δ*	DE	10*f*_0_	*C*_*i*_	$\bar {n}_{i}$	$\overline {OR}$	$\overline {FP}$	$\overline {dFP}$	$\overline {FPC}$
				(*sd*)	(*sd*)	(*sd*)	(*sd*)	(*sd*)
			$C_{{1}^{*}}$	30.7	45.7	0.5	0.4	0.2
				(7.9)	(6.7)	(0.3)	(0.3)	(0.9)
	50	8.5	$C_{{2}^{*}}$	21.8	19.4	5.4	7.5	20.7
				(5.9)	(5.0)	(2.1)	(4.4)	(20.9)
			*C*_3_	32.9	8.4	9.2	29.0	93.7
				(6.3)	(0.6)	(0.2)	(3.0)	(5.8)
			$C_{1}^{*}$	34.1	46.5	0.0	0.0	0.0
				(11.3)	(6.3)	(0.1)	(0.1)	(0.2)
1.5	100	8.5	$C_{2}^{*}$	56.2	22.5	1.5	1.8	1.6
				(10.3)	(2.7)	(0.67)	(9.0)	(2.0)
			*C*_3_	32.1	9.1	8.7	21.5	68.9
				(5.6)	(0.5)	(0.35)	(2.0)	(8.9)
			$C_{1}^{*}$	59.3	29.5	0.0	0.0	0.0
				(12.5)	(2.8)	(0.0)	(0.0)	(0)
	200	8.5	$C_{2}^{*}$	113.0	14.8	0.5	0.8	0.4
				(13.0)	(1.0)	(0.2)	(0.2)	(0.6)
			*C*_3_	25.3	8.0	6.0	9.9	14.9
				(6.5)	(0.5)	(0.9)	(1.8)	(8.2)
			$C_{1}^{*}$	22.3	86.3	0.0	0.0	0.0
				(9.2)	(17.1)	(0.1)	(0.0)	(0)
	50	8.0	$C_{2}^{*}$	27.8	42.0	1.4	1.5	5.8
				(6.9)	(9.2)	(2.0)	(3.3)	(18.0)
			*C*_3_	36.0	9.1	9.2	27.8	97.0
				(5.3)	(0.61)	(0.19)	(2.0)	(2.7)
			$C_{1}^{*}$	35.5	74.7	0.0	0.0	0.0
				(7.9)	(8.1)	(0.0)	(0.0)	(0)
2	100	8.0	$C_{2}^{*}$	63.1	37.3	0.4	0.4	0.3
				(7.9)	(3.1)	(0.2)	(0.2)	(0.7)
			*C*_3_	26.0	8.8	9.3	24.9	93.7
				(4.7)	(0.6)	(0.3)	(1.9)	(4.5)
			$C_{1}^{*}$	69.2	46.6	0.0	0.0	0.0
				(14.1)	(3.9)	(0.0)	(0.0)	(0)
	200	8.0	$C_{2}^{*}$	122.9	23.8	0.2	0.2	0.0
				(17.0)	(2.4)	(0.1)	(0.1)	(0.3)
			*C*_3_	13.1	8.9	8.1	13.0	58.0
				(17.3)	(2.4)	(2.0)	(4.1)	(23.1)
			$C_{1}^{*}$	18.9	191.2	0.0	0.0	0.0
				(6.6)	(22.7)	(0.0)	(0.0)	(0)
	50	7.2	$C_{2}^{*}$	31.5	99.9	0.29	0.4	2.1
				(4.9)	(16.1)	(1.2)	(2.6)	(14.1)
			*C*_3_	37.0	9.0	9.2	27.7	99.8
				(5.1)	(0.6)	(0.19)	(1.5)	(0.8)
			$C_{1}^{*}$	38.6	155.7	0.0	0.0	0.0
				(10.0)	(15.1)	(0.0)	(0.0)	(0)
3	100	7.1	$C_{2}^{*}$	61.3	83.4	0.0	0.0	0.0
				(10.0)	(6.6)	(0.1)	(0.0)	(0)
			*C*_3_	25.1	8.7	9.3	25.8	99.6
				(4.5)	(0.49)	(0.26)	(1.7)	(1.1)
			$C_{1}^{*}$	74.1	95.8	0	0	0.0
				(16.4)	(8.2)	(0)	(0)	(0)
	200	7.1	$C_{2}^{*}$	115.5	53.1	0.0	0.0	0.0
				(21.3)	(6.6)	(0.0)	(0.0)	(0)
			*C*_3_	16.1	12.5	8.3	16.4	86.0
				(24.8)	(11.9)	(3.2)	(6.8)	(33.6)

Evaluation of the second step of the ORdensity method with *α*=0.05. In the first two columns, delta (*Δ*) values and number of total simulated DE genes. In column 3, the 10×*f*_0_ values where *f*_0_ is the average proportion of permuted cases in sets *U*_*i*_. In column 4, the “*” indicates the clusters considered by the procedure. Columns 5–8 contain for each cluster: the mean number of genes ($\bar {n}_{i}$), the mean of OR values ($\overline {OR}$), the mean of *FP* values ($\overline {FP}$), the mean of *dFP* values ($\overline {dFP}$). In the last column the mean of False Positives genes per cluster in % ($\overline {FPC}$). Corresponding standard deviations are in brackets

With different samples sizes (*n*_*X*_=30,*n*_*Y*_=10) and even small sample sizes (*n*_*X*_=*n*_*Y*_=10), the average number of False Positive genes was again very small. The worst cases were obtained when only 50 simulated DE genes and *Δ*=1.5 were considered. In the majority of the other situations, the average number of False Positive genes was 0 or less than one (Table 4, Fig. 6 and in Additional file 1: Tables S18–S23, Figures S13–S18).

Moreover, for scenario 3, where DE genes were associated with different values of *Δ*_*g*_, observing the distribution of DE genes within each cluster in relation with their corresponding *Δ*_*g*_ values, we obtained that the most important DE genes, which are related to *Δ*_*g*_=3, are mainly in cluster 1, and that the DE genes included in cluster 3 are the less important since they are related most principally to *Δ*_*g*_=1.5 and never with *Δ*_*g*_=3. It is worth noting that even for a small number of samples (*n*_*X*_=*n*_*Y*_=10) similar results were obtained (Additional file 1: Tables S19, S22 and S23 last column and Figures S14, S17 and S18).

### Simulation study 2

#### General behavior

As in the above simulation study, the method identified correctly the DE genes. With the strong selection, again, no False Positive genes were obtained. With the more relaxed selection, the worst results were obtained with only 5 simulated DE genes. In the majority of situations the method only considers as DE genes those in cluster 1, with 0 or a very small number of False Positive genes.

#### Evaluation of the area under the ROC curve

The mean AUC values were very large and similar to those obtained by SAM and limma even when the sample sizes are small (Table 5, and Additional file 1: Tables S24 and S25).

**Table 5:** AUC mean values for Simulation study 1, scenario 1 and 100 runs. In first column: *n* indicates the number of DE gens; *Δ* the *Δ* values; A the ORdensity method; B the limma method and C the SAM method

	*n*_*X*_=*n*_*Y*_=30					
*n*		50			100	
*Δ*	1.5	2	3	1.5	2	3
A	0.998	0.998	0.997	0.998	0.997	0.995
	(0.002)	(0.001)	(0.002)	(0.001)	(0.002)	(0.003)
B	0.995	0.993	0.993	0.997	0.993	0.993
	(0.002)	(0.000)	(0.000)	(0.003)	(0.000)	(0.000)
C	0.994	0.993	0.993	0.996	0.993	0.993
	(0.003)	(0.000)	(0.000)	(0.002)	(0.000)	(0.000)
	*n*_*X*_=*n*_*Y*_=30			*n*_*X*_=30, *n*_*Y*_=10		
*n*		200			50	
*Δ*	1.5	2	3	1.5	2	3
A	0.996	0.997	0.993	0.974	0.996	0.998
	(0.000)	(0.000)	(0.000)	(0.001)	(0.001)	(0.001)
B	0.997	0.992	0.992	0.967	0.996	0.993
	(0.003)	(0.001)	(0.000)	(0.011)	(0.003)	(0.001)
C	0.998	0.992	0.992	0.940	0.994	0.993
	(0.002)	(0.001)	(0.000)	(0.016)	(0.003)	(0.001)
	*n*_*X*_=30, *n*_*Y*_=10					
*n*		100			200	
*Δ*	1.5	2	3	1.5	2	3
A	0.973	0.994	0.998	0.959	0.992	0.997
	(0.001)	(0.002)	(0.003)	(0.000)	(0.000)	(0.000)
B	0.981	0.997	0.993	0.993	0.998	0.992
	(0.006)	(0.003)	(0.000)	(0.002)	(0.002)	(0.000)
C	0.946	0.995	0.993	0.960	0.996	0.992
	(0.010)	(0.003)	(0.000)	(0.007)	(0.002)	(0.000)
	*n*_*X*_=*n*_*Y*_=10					
*n*		50			100	
*Δ*	1.5	2	3	1.5	2	3
A	0.925	0.974	0.998	0.921	0.971	0.997
	(0.014)	(0.011)	(0.011)	(0.010)	(0.001)	(0.008)
B	0.894	0.980	0.994	0.931	0.989	0.994
	(0.023)	(0.009)	(0.001)	(0.013)	(0.004)	(0.002)
C	0.859	0.959	0.994	0.865	0.960	0.994
	(0.023)	(0.015)	(0.001)	(0.016)	(0.009)	(0.002)
	*n*_*X*_=*n*_*Y*_=10					
*n*		200				
*Δ*	1.5	2	3			
A	0.900	0.956	0.996			
	(0.002)	(0.001)	(0.001)			
B	0.968	0.996	0.993			
	(0.007)	(0.001)	(0.003)			
C	0.876	0.974	0.995			
	(0.017)	(0.007)	(0.003)			

#### Detail evaluation of step 1

Concerning the results of the first step, we can observe that as a general behaviour, the method conrectly included the DE genes in the set of *potential* genes. With an equal number of samples (*n*_*X*_=*n*_*Y*_=30), the method included all the DE genes in the set of *potential* genes, being 99.8% the worst result in the case of 3 blocks (Table 6). When the number of samples in one condition is small (*n*_*X*_=30, *n*_*Y*_=10) and there is only one block of correlated genes, the same behaviour can be observed. With 2 or 3 blocks of correlated genes some of the DE can be missed, however, the proportion of DE genes that are actually in *S*_*α*_ is very large, being 87.5% in the worst case (Additional file 1: Table 26, Figure 19).

**Table 6:** Simulation study 2 with *n*_*X*_=*n*_*Y*_=30 and 100 runs

Number of		1 block			2 blocks		
DE genes		*α*			*α*		
per block		0.1	0.05	0.01	0.1	0.05	0.01
5	$\hat {p}_{m}$	0	0	0	0	0	0
	%	100 (0)	100 (0)	100 (0)	100 (0)	100 (0)	100 (0)
20	$\hat {p}_{m}$	0	0	0	0	0	0
	%	100 (0)	100 (0)	100 (0)	100 (0)	100 (0)	100 (0)
100	$\hat {p}_{m}$	0	0	0	0	0	0
	%	100 (0)	100 (0)	100 (0)	100 (0)	100 (0)	100 (0)
Number of		3 blocks					
DE genes		*α*					
per block		0.1	0.05	0.01			
5	$\hat {p}_{m}$	0	0	0			
	%	100 (0)	100 (0)	100 (0)			
20	$\hat {p}_{m}$	0	0	0.01			
	%	100	100	99.9 (0.7)			
100	$\hat {p}_{m}$	0	0.01	0.04			
	%	100 (0)	100.0 (0.0)	99.8 (1.9)			

Evaluation of the first step of the ORdensity method using different values of *α*. The Table shows the estimated probability, $\hat {p}_{m}$, of no considering as a *potential* DE gene at least one gene that it really is, and the mean proportion of DE genes (row named “%”) that the procedure considered as *potential* DE genes. Corresponding standard deviations are in brackets

#### Detail evaluation of step 2

**Fig. 7:**
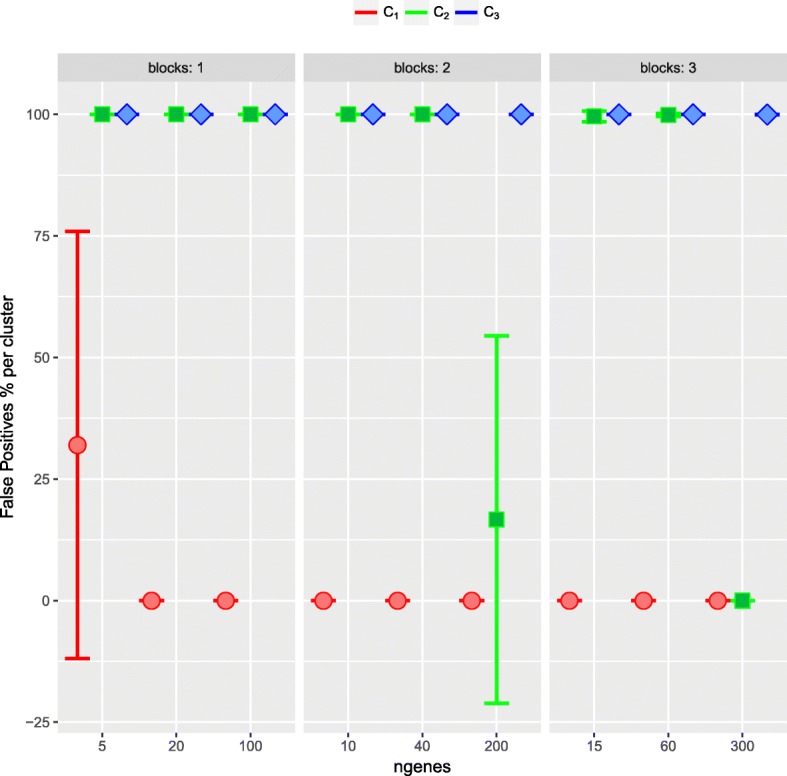
Simulation study 2 with *n*_*X*_=*n*_*Y*_=30 and 100 runs. Evaluation of the second step of the ORdensity method with *α*=0.05. In *x* axis number of DE genes; in *y* axis the mean of False Positives genes per cluster in % ($\overline {FPC}$). In red cluster 1 (C_1_), in green cluster 2 (C_2_) and in blue cluster 3 (C_3_)

As a general comment, one can see a strong correlation/correspondence between being a cluster with high *OR* and low false positiveness in the neighbourhood (*FP*, *dFP*), along with a small number of False Positive genes in the cluster. This holds for a different number of blocks and a different number of genes in the blocks. With *n*_*X*_=*n*_*Y*_=30 and one block (Table 7, Fig. 7), the worst results were obtained for 5 simulated DE genes. In this case, the method only considered those in cluster 1 as DE genes, but the size of cluster 1 varied between 5 and 58 genes. This high variability in the number of genes in cluster 1 is probably due to always considering 3 clusters. For 20 or 100 simulated DE genes, again the procedure only considers genes in cluster 1 and not one False Positive gene was detected. For two blocks, with 5 and 20, again only cluster 1 was considered and no False Positives were obtained. With 100 simulated DE genes in each block, clusters 1 and 2 were considered by the method with 0 and 15.9 False Positives genes, respectively. With three blocks, independently of the number of simulated DE genes, no False Positives were found. Similar results were obtained for *n*_*X*_=30,*n*_*Y*_=10 (Additional file 1: Table 27, Figure 20).

**Table 7:** Simulation study 2, with *n*_*X*_=*n*_*Y*_=30 and 100 runs

B	DE	10*f*_0_	*C*_*i*_	$\bar {n}_{i}$	$\overline {OR}$	$\overline {FP}$	$\overline {dFP}$	$\overline {FPC}$
				(*sd*)	(*sd*)	(*sd*)	(*sd*)	(*sd*)
			$C_{1}^{*}$	27.9	160.4	6.6	8.1	32.0
				(31.2)	(102.2)	(1.1)	(10.8)	(43.9)
	5	9.1	*C*_2_	92.1	10.1	9.0	24.1	100
				(25.5)	(0.8)	(0.4)	(4.0)	(0)
			*C*_3_	82.5	7.8	9.4	41.6	100
				(17.9)	(0.3)	(0.3)	(2.2)	(0)
			$C_{1}^{*}$	20.0	233.0	0.0	0.0	0.0
				(0.0)	(35.1)	(0.0)	(0.0)	(0)
1	20	9.1	*C*_2_	96.5	10.4	9.0	22.0	100
				(12.7)	(0.5)	(0.1)	(1.5)	(0)
			*C*_3_	90.1	7.7	9.2	41.1	100
				(14.2)	(0.2)	(0.1)	(1.4)	(0)
			$C_{1}^{*}$	100.0	209.5	0.0	0.0	0.0
				(0.0)	(32.9)	(0.0)	(0.0)	(0)
	100	8.9	*C*_2_	73.7	10.8	9.1	20.7	100
				(10.9)	(0.59)	(0.2)	(1.5)	(0)
			*C*_3_	69.1	8.2	9.2	37.5	100
				(11.2)	(0.25)	(0.2)	(1.6)	(0)
			$C_{1}^{*}$	9.9	119.7	1.0	0.2	0.0
				(0.6)	(16.9)	(0.2)	(0.0)	(0.0)
	10	9.1	*C*_2_	102.2	10.5	9.0	22.1	99.9
				(14.2)	(0.7)	(0.1)	(1.5)	(0.7)
			*C*_3_	94.2	7.7	9.2	41.7	100
				(13.2)	(0.2)	(0.1)	(1.4)	(0.0)
			$C_{1}^{*}$	40.0	118.6	0.0	0.0	0.0
				(0.1)	(20.0)	(0.0)	(0.0)	(0.0)
2	40	9.1	*C*_2_	97.0	10.3	9.1	22.2	100
				(12.0)	(0.5)	(0.1)	(0.4)	(0.0)
			*C*_3_	87.4	7.7	9.2	41.2	100
				(12.9)	(0.2)	(0.1)	(1.2)	(0.0)
			$C_{1}^{*}$	115.9	120.1	0.0	0.0	0.0
				(38.9)	(18.9)	(0.0)	(0.0)	(0.0)
	200	8.6	$C_{2}^{*}$	95.3	72.7	1.6	3.7	16.7
				(15.5)	(31.3)	(3.5)	(8.2)	(37.8)
			*C*_3_	118.5	8.9	9.3	31.2	100
				(28.3)	(0.5)	(0.1)	(3.2)	(0.0)
			$C_{1}^{*}$	14.6	86.2	0.26	0.09	0.0
				(1.14)	(14.1)	(0.31)	(0.11)	(0)
	15	9.0	*C*_2_	103.9	9.0	8.4	22.1	99.6
				(11.8)	(0.13)	(0.48)	(1.5)	(1.1)
			*C*_3_	92.8	7.2	9.2	41.6	100
				(10.7)	(0.77)	(0.13)	(1.4)	(0.0)
			$C_{1}^{*}$	59.9	81.2	0.03	0.03	0.0
				(0.29)	(12.1)	(0.07)	(0.06)	(0)
3	60	8.9	*C*_2_	93.8	10.3	9.1	22.3	99.9
				(12.3)	(0.43)	(0.13)	(1.4)	(0.3)
			*C*_3_	87.2	7.7	9.2	41.3	100
				(11.4)	(0.16)	(0.13)	(1.3)	(0.0)
			$C_{1}^{*}$	142.4	81.0	0.00	0.00	0.0
				(31.0)	(13.7)	(0.00)	(0.00)	(0)
	300	8.4	$C_{2}^{*}$	157.5	54.6	0.03	0.04	0.0
				(31.0)	(11.1)	(0.06)	(0.09)	(0)
			*C*_3_	119.2	8.9	9.4	30.3	99.9
				(11.4)	(0.28)	(0.10)	(0.96)	(0.02)

Evaluation of the second step of the ORdensity method with *α*=0.05. In the first column number of blocks. In column 2, the number of total simulated DE genes. In column 3, the 10×*f*_0_ values where *f*_0_ is the average proportion of permuted cases in sets *U*_*i*_. In column 4, the “*” indicates the clusters considered by the procedure. Columns 5–8 contain for each cluster: the mean number of genes ($\bar {n}_{i}$), the mean of OR values ($\overline {OR}$), the mean of *FP* values ($\overline {FP}$), the mean of *dFP* values ($\overline {dFP}$). In the last column the mean of False Positives genes per cluster in % ($\overline {FPC}$). Corresponding standard deviations are in brackets

#### Evaluation of the area under the ROC curve

For *n*_*X*_=*n*_*Y*_=30, the mean AUC values were very large and somewhat better than those obtained with limma and SAM, especially for one and two blocks (Table 8). Similar results were found for *n*_*X*_=30, *n*_*Y*_=10.

**Table 8:** AUC mean values for Simulation study 2, with *n*_*X*_=*n*_*Y*_=30 and 100 runs

*n*_*X*_=*n*_*Y*_=30						
	1 block			2 blocks		
Nb. DE genes	5	20	100	5	20	50
ORdensity	0.995	0.995	0.980	0.993	0.998	0.985
	(0.000)	(0.000)	(0.000)	(0.000)	(0.000)	(0.000)
limma	0.701	0.854	0.903	0.796	0.789	0.917
	(0.0004)	(0.001)	(0.001)	(0.000)	(0.001)	(0.045)
SAM	0.701	0.854	0.902	0.796	0.789	0.918
	(0.001)	(0.001)	(0.00002)	(0.000)	(0.0003)	(0.045)
	3 block					
Nb. DE genes	5	20	50			
ORdensity	0.983	0.997	0.990			
	(0.002)	(0.000)	(0.000)			
limma	0.982	0.997	0.990			
	(0.001)	(0.003)	(0.069)			
SAM	0.825	0.994	0.876			
	(0.000)	(0.003)	(0.070)			

In summary, the results with simulated data showed that ORdensity correctly detects the DE genes and is competitive with other well-known methods.

### Actual data: lymphoma, Golub, colon and prostate cancer data sets

For the three procedures, the number of genes selected under the standard criterion varies, being much larger for SAM and limma.

For the lymphoma data set (1095 genes), the ORdensity procedure selected 96 *potential* DE genes (*#S*_0.05_=96), distributed in two clusters with 19 and 77 genes, respectively. However, the strong selection could not be used since no gene had *FP* and *dFP* values equal to 0. The limma method produced a list with 24 and 88 genes using Bonferroni and BH procedures, respectively. The SAM procedure produced a list containing 64 genes. When the Golub data set (with 7129 genes) was considered, the procedure selected 556 *potential* DE genes, distributed in two groups with 291 and 265 genes, respectively. Among the genes in cluster 1, only 4 had *FP* and *dFP* values equal to zero. Thus, with the ORdensity, the most restricted criterion gave a list with only 4 genes and the relaxed criterion gave a list with 291 genes. The limma method produced a list with 193 and 938 genes using, respectively, Bonferroni and BH methods, and the SAM procedure produced a list with 403 genes.

For the colon data set (6000 genes), the ORdensity procedure selected 186 genes as DE *potential* genes (*#S*_0.05_=186), distributed in three clusters with 59, 88 and 39 genes, respectively. Among the 59 genes of cluster 1, twelve of them had no false positive permuted cases in their neighbourhood, with *FP* and *dFP* values equal to zero. Thus, with the ORdensity, the most restricted criterion gave a list with only 12 genes and the relaxed criterion gave a list with 59 genes. The limma method produced a list with 49 and 366 genes using Bonferroni and BH, respectively, and the SAM procedure produced a list containing 166 genes.

In the case of the prostate data set (12626 genes), 1531 *potential* DE genes (*#S*_0.05_=1531) were detected belonging to two clusters with 990 and 541 genes, respectively. Out of the 990 genes in cluster 1, 131 had no false positive permuted cases in their neighbourhood, with *FP* and *dFP* values equal to zero. Different number of selected genes were considered: the 131 produced by the more restricted ORdensity; the 990 for the relaxed ORdensity; the 1531 total candidate genes; the 263 and 2684 selected by limma rule under Bonferroni and BH, respectively, and the 3322 genes selected under SAM procedure.

The results obtained with these actual data sets are shown in Tables 9, 10, 11 and 12, respectively. The leave-one-out cross-validation correct classification rate (rows I in Tables 9, 10, 11 and 12) indicates that ORdensity does not lead to overfitting, and can achieve the objectives of reducing the set of selected genes and reaching high leave-one-out cross-validation correct classification accuracy rates. With the lymphoma data set, the ORdensity, with the 19 genes selected, reached 100% of leave-one-out cross-validation correct classification rate and this result was matched by SAM and limma using 19 or 24 genes, respectively. With the Golub data set and using only 4 genes, a correct classification rate of 90.28% was reached and with the 291 genes selected by the relaxed selection the maximum value of 97.22% was obtained. With limma or SAM, using the first 4 selected genes, the classification rate was improved (97.22 *versus* 90.28), but using limma or SAM there was not any objective criterion to select 4 genes. A similar situation happened with the colon and prostate data sets.

**Table 9:** Lymphoma cancer data set

		Ns				
		19	24	64	88	96
I	ORdensity	**100**	100	100	100	100
	limma	95.24	**100**	100	**100**	-
	SAM	100	100	**100**	-	-
II	A vs B	13	17	14	17	-
	A vs C	14	16	42	63	-
	B vs C	17	23	58	-	-
III	ORdensity	10.2	13.2	40.4	61.4	65.3
		(1.48)	(2.84)	(2.01)	(2.27)	(3.13)
	limma	7.9	9.7	27.4	36.7	-
		(0.87)	(1.57)	(2.17)	(1.34)	-
	SAM	14.2	17.3	51.4	-	-
		(2.82)	(3.97)	(8.85)	-	-

Results for different number (Ns) of selected genes: 19 with ORdensity relaxed selection; 24 with limma and Bonferroni; 64 with SAM; 88 with limma and BH, and 96 total *potential* DE genes. Rows I present the leave-one-out cross-validation correct classification rate. In bold, the results for the genes selected under standard criteria for ORdensity, limma and SAM procedures; in rows II, number of common selected genes between the ORdensity, limma and SAM approaches; in rows III, mean and standard deviation (in brackets) of the number of genes that for 10 subsamples were always kept selected

**Table 10:** Golub cancer data set

		Ns		
		4	193	291
I	ORdensity (A)	**90.28**	97.22	**97.22**
	limma (B)	97.22	**97.22**	97.22
	SAM (C)	97.22	97.22	97.22
II	A vs B	1	127	206
	A vs C	1	130	213
	B vs C	4	175	265
III	ORdensity	0.5 (0.71)	150.4 (3.89)	241.8 (6.94)
	limma	0	12.3 (2.26)	4.3 (1.83)
	SAM	3.3 (0.48)	151.8 (24.18)	220.7 (35.88)
		Ns		
		403	556	938
I	ORdensity (A)	97.22	97.22	-
	limma (B)	97.22	97.22	**97.22**
	SAM (C)	**97.22**	-	-
II	A vs B	274	358	-
	A vs C	280	-	-
	B vs C	368	-	-
III	ORdensity	334.1 (6.03)	473.3 (9.56)	-
	limma	029.2 (2.44)	55.5 (3.10)	149.3 (6.36)
	SAM	316.3 (50.06)	-	-

Results for different number (Ns) of selected genes: 4 with ORdensity strong selection; 193 with limma and Bonferroni; 291 with ORdensity relaxed selection; 403 with SAM; 556 total *potential* DE genes and 938 with limma and BH. Rows I present the leave-one-out cross-validation correct classification rate. In bold, the results for the genes selected under the standard criteria for ORdensity, limma and SAM procedures; in rows II, number of common selected genes between the ORdensity, limma and SAM approaches; in rows III, mean and standard deviation (in brackets) of the number of genes that for 10 subsamples were always kept selected

**Table 11:** Colon cancer data set

		Ns		
		12	49	59
I	ORdensity (A)	**88.71**	90.32	**90.32**
	limma (B)	91.94	**88.71**	88.71
	SAM (C)	85.48	88.71	88.71
II	A vs B	7	32	38
	A vs C	0	14	22
	B vs C	2	26	29
III	ORdensity	7.5 (1.27)	35.4 (4.09)	43.0 (3.56)
	limma	0.1 (0.32)	3.5 (0.85)	4.1 (0.57)
	SAM	1.8 (1.14)	23.7 (2.98)	29.4 (3.57)
		Ns		
		166	186	366
I	ORdensity (A)	87.10	87.10	-
	limma (B)	87.10	87.10	**87.10**
	SAM (C)	**87.10**	-	-
II	A vs B	119	134	-
	A vs C	118	-	-
	B vs C	155	-	-
III	ORdensity	127.2 (4.49)	142.5 (4.84)	-
	limma	13.1 (1.37)	16.7 (2.21)	72.1 (4.72)
	SAM	136.7 (5.23)	-	-

Results for different number (Ns) of selected genes: 12 with ORdensity strong selection; 49 with limma and Bonferroni; 59 with ORdensity relaxed selection; 166 with SAM; 186 total *potential* DE genes and 366 with limma and BH. Rows I present the leave-one-out cross-validation correct classification rate. In bold, the results for the genes selected under standard criteria for ORdensity, limma and SAM procedures; in rows II, number of common selected genes between the ORdensity, limma and SAM approaches. In rows III, mean and standard deviation (in brackets) of the number of genes that for 10 subsamples were always kept selected

**Table 12:** Prostate cancer data set

		Ns		
		131	263	990
I	ORdensity (A)	**75.49**	74.51	**70.59**
	limma (B)	86.27	**82.35**	71.57
	SAM (C)	82.35	76.47	70.59
II	A vs B	64	170	691
	A vs C	64	159	746
	B vs C	54	211	791
III	ORdensity	72.4 (7.12)	184.1 (23.56)	787.6 (29.40)
	limma	108.6 (4.99)	220.2 (10.07)	827.2 (39.17)
	SAM	0.7 (0.67)	4.4 (0.84)	66.1 (4.38)
		Ns		
		1531	2684	3322
I	ORdensity (A)	66.67	-	-
	limma (B)	70.59	**68.63**	-
	SAM (C)	70.59	66.67	**65.69**
II	A vs B	961	-	-
	A vs C	1035	-	-
	B vs C	1321	2422	-
III	ORdensity	1215.4 (99.66)	-	-
	limma	1297.9 (50.64)	2299.5 (86.19)	-
	SAM	165.1 (6.15)	563 (19.87)	862.2 (19.41)

Results for different number (Ns) of selected genes: 131 with ORdensity strong selection; 263 with limma and Bonferroni; 990 with ORdensity relaxed selection; 1531 total *potential* DE genes; 2684 with limma and BH, and 3322 with SAM. Rows I present the leave-one-out cross-validation correct classification rate. In bold, the results for the genes selected under standard criteria for ORdensity, limma and SAM procedures; in rows II, number of common selected genes between the ORdensity, limma and SAM approaches; in rows III, mean and standard deviation (in brackets) of the number of genes that for 10 subsamples were always kept selected

As can be observed (rows II in Tables 9, 10, 11 and 12), the three methods only share some of the genes in their respective lists, as is usual in these type of procedures. Furthermore, the method with which our analysis shares more genes varies according to each data set.

These results indicated that ORdensity returns a small number of crucial genes, that are strongly related to the disease, since the values of leave-one-out cross-validation correct classification rates are large. As a general trend, SAM and limma consider a larger number of DE genes, but nevertheless it does not guarantee to obtain better leave-one-out cross-validation correct classification rates.

It is important to note that ORdensity identifies several genes, not detected by the other methods, that are biologically relevant. For instance, consider the strong selection with the leukemia data set. Interestingly, genes selected only by our method could be fundamental to explain some traits of the leukemia. For example, our method recognizes genes that codify for cyclin D2 (protein involved in cell cycle), neprilysin (an enzyme common in acute lymphoblastic leukemia), a protein-tyrosine phosphatase of T-cells and a protein similar to phorbolin-1 (that can be expressed in leukocytes).

Finally, we evaluated the stability of the procedure in order to assess how often a gene, selected as DE with the original sample, was selected again when a fraction of the 20% of the original data was eliminated at random. In order to mitigate any effect of selection bias, the process was repeated 10 times. Note that (rows III in Tables 9, 10, 11 and 12) ORdensity procedure was, in general, the method that best kept a coherent list with DE genes.

From a biomedical point of view, all the above results indicated that our method is very valuable to detect a small group of genes with a large effect in a particular disease. Therefore, this information can be used to develop new in vivo and in vitro studies.

## Discussion

In this paper, a novel procedure, ORdensity, is proposed for the detection of differentially expressed genes. The proposed method is not a gene-by-gene procedure, and it takes into account the relationships between genes. The procedure obtains a ranking of importance of genes based in three measures, which reflect if the differences between quantiles for a gene under the two considered experimental conditions are important enough in order to consider the gene as a DE gene and if, in its neighbourhood, there are permuted false positives or not. We have presented an exhaustive evaluation of the performance of ORdensity using simulated microarray data, and results indicated that the new method correctly detects the DE genes. Furthermore, the simulation study showed that the procedure is useful even with small samples and it is competitive with other well established procedures, such as SAM and limma. The results, obtained with actual cancer microarray data sets, showed that ORdensity is very useful to obtain a small number of DE genes with high correct classification rate by the leave-one-out cross-validation approach and it is, in general, more stable than other well accepted procedures when the original sample is substituted with a subsample.

The main advantages of the proposed method are, therefore, that it returns very small sets of genes that retain a high predictive accuracy; the selected gene list is stable; it avoids the classical multiple comparison restrictions; as it takes into consideration the tails of distributions, it can detect outlying genes that only exhibit differential qualities in the tails, and, moreover, as we can cluster the genes using the three discriminative values OR, FP and dFP, different patterns of genes can be obtained. The results generated by our new procedure could be of extreme interest to biomedical research, because they can focus on a short, but crucial number of genes. Thus, these small numbers of genes could act as the corner stones to understand the origin and development of several serious diseases. The idea that lies beneath the proposed methodology seems applicable to RNA-Sequencing data. However, although it is usual to model RNA-Seq data as both negative binomial distribution and as normal distribution by ln-transforming normalized count data, because the properties of RNA-Seq data have not yet been fully established, additional research is needed.

## Conclusions

Here we present a new method to identify differentially expressed genes that avoids losing sensitivity due to correction by multiple comparisons. This method is able to identify the nucleus of the genes that are candidates to explain a particular disease or pathology. With this information, further biomedical studies can be developed, focusing the attention in these candidate genes.

## Additional file

Additional file 1 This file contains Tables 13-27 and Figures 8-20 cited in the text. (PDF 533 kb)

## Abbreviations

ALL: Acute lymphoblastic leukemia
AML: Acute myelooblastic leukemia
AUC: Area under the ROC curve
BH: Benjamini and Hochberg adjustment method
DE: Differentially expressed
DLBCL: Large B cell lymphoma
dFP: Average density of false positive permuted cases in 10-NN
FDR: False discovery Rate
FP: Average number of false positive permuted cases in 10-NN
FPC: Percentage of false positive genes per cluster
FWER: Family wise error rate
limma: Linear models with empirical Bayes statistic
OR: Outlier robust index
PAM: Partition around Medoids clustering method
ROC: Receiver operating characteristic
SAM: The significant analysis of microarrays
WDB-discriminant: weighted distance based discriminant
