# Supplementary material for: Identification of differentially expressed genes by means of outlier detection
Source: BMC Bioinformatics. 2018 Sep 10;19:317. doi: 10.1186/s12859-018-2318-8 (PMC6131896; doi:10.1186/s12859-018-2318-8)
Supplement: Supplementary file 1 — This file contains Tables 13-27 and Figures 8-20 cited in the text. (PDF 533 kb) [file 12859_2018_2318_MOESM1_ESM.pdf]

Additional File 1

Below are Tables 13 − 27 and Figures 8 − 20 related to the results of different scenarios established in the Experimental Setup Section.

TABLES 13 − 27

Table 13 **Simulation study 1, scenario 2 with $n_X = n_Y = 30$ and 100 runs. Evaluation of the first step of the ORdensity method using different values of α. The Table shows the estimated probability, $\hat{p}_m$, of no considering as a potential DE gene at least one gene that it really is, and the mean proportion of DE genes (row named "%") that the procedure considered as potential DE genes.**

Nb. DE genes		$\Delta = 1.5$			$\Delta = 2$			$\Delta = 3$		
		α			α			α		
		0.1	0.05	0.01	0.1	0.05	0.01	0.1	0.05	0.01
50	$\hat{p}_m$	0.00	0.08	0.66	0.00	0.00	0.00	0.01	0.00	0.00
	%	100	99.84 (0.55)	98.00 (2.00)	100	100	99.98 (0.20)	100	100	100
100	$\hat{p}_m$	0.06	0.26	0.98	0.00	0.00	0.02	0.00	0.00	0.00
	%	99.94 (0.24)	99.67 (0.65)	96.37 (2.12)	100	100	99.98 (0.14)	100	100	100
200	$\hat{p}_m$	0.47	0.95	1.00	0.01	0.03	0.79	0.00	0.00	0.00
	%	99.69 (0.37)	97.48 (0.90)	85.24 (2.70)	99.99 (0.05)	99.98 (0.09)	99.27 (0.54)	100	100	100

Table 14 **Simulation study 1, scenario 3 with $n_X = n_Y = 30$ and 100 runs. Evaluation of the first step of the ORdensity method using different values of α. The Table shows the estimated probability, $\hat{p}_m$, of no considering as a potential DE gene at least one gene that it really is; the mean proportion of DE genes (row named "%") that the procedure considered as potential DE genes, and distribution of truly DE genes that are not considered as a potential DE gene in relation with their value of Δ_g taken by chance in $\{1.5, 2, 3\}$.**

Number of DE genes		α		
		0.1	0.05	0.01
50	$\hat{p}_m$	0.01	0.03	0.30
	%	99.98 (0.20)	99.94 (0.34)	99.32 (1.11)
	$(\Delta_g = 1.5, \Delta_g = 2, \Delta_g = 3)$	(100, 0, 0)	(100, 0, 0)	(100, 0, 0)
100	$\hat{p}_m$	0.02	0.10	0.70
	%	99.98 (0.14)	99.89 (0.35)	98.91 (1.02)
	$(\Delta_g = 1.5, \Delta_g = 2, \Delta_g = 3)$	(100, 0, 0)	(100, 0, 0)	(99.3, 0.7, 0)
200	$\hat{p}_m$	0.21	0.62	1.00
	%	99.87 (0.26)	99.50 (0.53)	95.09 (1.50)
	$(\Delta_g = 1.5, \Delta_g = 2, \Delta_g = 3)$	(100, 0, 0)	(100, 0, 0)	(96.3, 3.7, 0)

Table 15 Simulation study 1, scenario 2 with $n_X = 30$, $n_Y = 10$ and 100 runs. Evaluation of the first step of the ORdensity method using different values of α. The Table shows the estimated probability, $\hat{p}_m$, of no consider as a potential DE gene at least one gene that it really is, and the mean proportion of DE genes (row named "%") that the procedure considered as potential DE genes.

Nb. DE genes		$\Delta = 1.5$			$\Delta = 2$			$\Delta = 3$		
		α			α			α		
		0.1	0.05	0.01	0.1	0.05	0.01	0.1	0.05	0.01
50	$\hat{p}_m$	0.70	0.96	1	0.06	0.22	0.88	0.00	0.00	0.00
	%	97.6 (2.1)	93.5 (3.3)	74.7 (5.3)	99.9 (0.5)	99.5 (0.9)	95.6 (3.1)	100	100	100
100	$\hat{p}_m$	0.98	1	1	0.09	0.50	1	0.00	0.00	0.10
	%	96.5 (1.7)	90.9 (2.7)	66.4 (4.7)	99.9 (0.4)	99.2 (1.0)	92.4 (3.0)	100	100	99.9 (0.3)
200	$\hat{p}_m$	1	1	1	0.88	1	1	0	0.02	0.80
	%	90.1 (2.0)	77.6 (2.6)	43.4 (3.7)	98.9 (0.8)	95.5 (1.7)	74.7 (3.9)	100	99.9 (0.1)	99.0 (0.8)

Table 16 Simulation study 1, scenario 3 with $n_X = 30$, $n_Y = 10$ and 100 runs. Evaluation of the first step of the ORdensity method using different values of α. The Table shows the estimated probability, $\hat{p}_m$, of no considering as a potential DE gene at least one gene that it really is; the mean proportion of DE genes (row named "%") that the procedure considered as potential DE genes, and distribution of truly DE genes that are not considered as a potential DE gene in relation with their value of Δ_g taken by chance in $\{1.5, 2, 3\}$.

Nb. of DE genes		α		
		0.1	0.05	0.01
	$\hat{p}_m$	0.38	0.75	1
50	%	99.1 (1.2)	97.7 (1.9)	99.8 (3.8)
	$(\Delta_g = 1.5, \Delta_g = 2, \Delta_g = 3)$	(97.4, 2.6, 0)	(97.3, 2.7, 0)	(85.9, 14.1, 0)
	$\hat{p}_m$	0.73	0.95	1
100	%	98.8 (1.0)	96.8 (1.7)	86.6 (3.4)
	$(\Delta_g = 1.5, \Delta_g = 2, \Delta_g = 3)$	(95.9, 4.1, 0)	(91.0, 9.0, 0)	(82.2, 17.5, 0.3)
	$\hat{p}_m$	1	1	1
200	%	96.2 (1.4)	91.0 (2.2)	71.1 (3.4)
	$(\Delta_g = 1.5, \Delta_g = 2, \Delta_g = 3)$	(92.7, 7.3, 0)	(86.7, 13.1, 0.2)	(96.3, 3.7, 0)

Table 17 Simulation study 1, scenario 3 with $n_X = n_Y = 10$ and 100 runs. Evaluation of the first step of the ORdensity method using different values of α. The Table shows the estimated probability, $\hat{p}_m$, of no considering as a potential DE gene at least one gene that it really is; the mean proportion of DE genes (row named "%") that the procedure considered as potential DE genes, and distribution of truly DE genes that are not considered as a potential DE gene in relation with their value of Δ_g taken by chance in $\{1.5, 2, 3\}$.

Nb. DE genes		α		
		0.1	0.05	0.01
	$\hat{p}_m$	0.91	0.98	1
50	%	95.3 (3.1)	91.0 (4.1)	73.1 (6.4)
	$(\Delta_g = 1.5, \Delta_g = 2, \Delta_g = 3)$	(89.5, 10.5, 0)	(83.7, 16.3, 0)	(67.0, 30.7, 0.0)
	$\hat{p}_m$	1	1	1
100	%	94.3 (2.3)	87.6 (3.2)	65.8 (5.4)
	$(\Delta_g = 1.5, \Delta_g = 2, \Delta_g = 3)$	(83.9, 15.9, 0.2)	(79.1, 20.3, 0.6)	(63.7, 32.1, 4.2)
	$\hat{p}_m$	1	1	1
200	%	87.0 (2.1)	75.8 (2.8)	51.5 (3.8)
	$(\Delta_g = 1.5, \Delta_g = 2, \Delta_g = 3)$	(76.5, 23.0, 0.4)	(67.4, 30.7, 1.9)	(52.4, 37.6, 10.0)

Table 18 Simulation study 1, scenario 2 with $n_X = n_Y = 30$ and 100 runs. Evaluation of the second step of the ORdensity method with $\alpha = 0.05$. In the first two columns, the delta Δ values and number of total simulated DE genes. In column 3, the $10 \times f_0$ values where f_0 is the average proportion of permuted cases in sets U_i. In column 4, the "*" indicates the clusters considered by the procedure. Columns 5-8 contain for each cluster: the mean number of genes $(n^{-}{}_i)$, the mean of OR values $(\overline{OR})$, the mean of FP values $(\overline{FP})$, the mean of dFP values $(\overline{dFP})$. In the last column the mean of False Positive genes per cluster in % $(\overline{FPC})$. Corresponding standard deviations are in brackets.

Δ	DE	$10f_0$	C_i	$\bar{n}_i$ (sd)	$\overline{OR}$ (sd)	$\overline{FP}$ (sd)	$\overline{dFP}$ (sd)	$\overline{FPC}$ (sd)
1.5	50	8.7	C_1^*	24.6 (9.2)	51.4 (10.0)	0.2 (0.2)	0.1 (0.2)	0.0 (0.5)
			C_2^*	23.0 (6.7)	23.1 (5.2)	3.4 (2.0)	3.3 (2.6)	6.4 (11.3)
			C_3	27.6 (4.7)	8.7 (0.8)	9.2 (0.3)	23.6 (2.7)	87.9 (8.0)
	100	8.1	C_1^*	31.8 (11.1)	50.3 (6.6)	0.0 (0.0)	0.0 (0.0)	0.0 (0)
			C_2^*	59.6 (10.8)	23.5 (2.7)	1.1 (0.7)	1.1 (0.7)	0.3 (1.4)
			C_3	24.6 (9.2)	9.1 (0.7)	8.7 (0.4)	18.3 (2.2)	65.8 (10.7)
	200	7.1	C_1^*	32.5 (14.1)	33.5 (3.3)	0.0 (0.0)	0.0 (0.0)	0.0 (0)
			C_2^*	116.8(13.4)	16.1 (1.2)	0.4 (0.1)	0.5 (0.2)	0.3 (0.5)
			C_3	21.1 (6.3)	8.1 (0.6)	6.2 (1.1)	8.8 (2.0)	17.3 (10.1)
2	50	8.7	C_1^*	17.7 (6.1)	97.7 (14.9)	0.0 (0.0)	0.0 (0.0)	0.0 (0)
			C_2^*	31.6 (5.5)	46.4 (5.3)	0.7 (0.5)	0.4 (0.3)	0.4 (1.3)
			C_3	26.9 (4.2)	8.9 (0.6)	9.3 (0.2)	24.1 (1.9)	95.9 (3.8)
	100	8.1	C_1^*	33.7 (8.9)	82.8 (10.1)	0.0 (0.0)	0.0 (0.0)	0.0 (0)
			C_2^*	65.1 (8.7)	39.5 (3.1)	0.3 (0.1)	0.2 (0.1)	0.3 (0.6)
			C_3	19.5 (3.5)	8.8 (0.7)	9.3 (0.3)	21.6 (2.0)	92.9 (5.9)
	200	7.1	C_1^*	67.7 (17.6)	54.2 (5.8)	0.0 (0.0)	0.0 (0.0)	0.0 (0)
			C_2^*	115.3 (22.0)	27.2 (4.7)	0.1 (0.1)	0.1 (0.1)	0.1 (0.3)
			C_3	21.4 (30.2)	10.6 (4.9)	7.2 (3.2)	10.6 (5.4)	54.2 (30.3)
3	50	8.7	C_1^*	17.6 (6.1)	205.9 (29.4)	0.0 (0.0)	0.0 (0.0)	0.0 (0)
			C_2^*	32.5 (5.5)	101.9 (12.9)	0.2 (0.9)	0.2 (1.7)	1.0 (10.0)
			C_3	24.8 (4.4)	8.6 (0.6)	9.3 (0.2)	24.6 (2.0)	99.8(0.8)
	100	8.1	C_1^*	36.3 (8.3)	174.7 (17.6)	0.0 (0.0)	0.0 (0.0)	0.0 (0)
			C_2^*	63.6 (8.3)	87.2 (7.1)	0.0 (0.0)	0.0 (0.0)	0.0 (0.2)
			C_3	18.1 (3.6)	8.5 (0.7)	9.4 (0.3)	22.8 (1.9)	99.4 (1.9)
	200	7.1	C_1^*	70.0 (17.2)	115.0 (12.4)	0.0 (0.0)	0.0 (0.0)	0.0 (0)
			C_2^*	120.7 (21.3)	59.2 (8.3)	0.0 (0.0)	0.0 (0.0)	0.0 (0.0)
			C_3	14.8 (25.2)	12.2 (12.5)	8.4 (3.0)	14.8 (5.7)	88.4 (31.3)

Table 19 Simulation study 1, scenario 3 with $n_X = n_Y = 30$ and 100 runs. Evaluation of the second step of the ORdensity method with $\alpha = 0.05$. In the first column the number of total simulated DE genes. In column 2, the $10 \times f_0$ values where f_0 is the average proportion of permuted cases in sets U_i. In column 4, the "*" indicates the clusters considered by the procedure. In column 3, the "*" indicates the clusters considered by the procedure. Columns 4-7 contain for each cluster: the mean number of genes $(n^{-}{}_i)$, the mean of OR values $(\overline{OR})$, the mean of FP values $(\overline{FP})$, the mean of dFP values $(\overline{dFP})$. In column 8 the mean of False Positive genes per cluster in % $(\overline{FPC})$. Corresponding standard deviations are in brackets. In the last column, distribution of DE genes in accordance with their Δ_g value that they have taken by chance in $\{1.5, 2, 3\}$.

DE	$10f_0$	C_i	$\bar{n}_i$ (sd)	$\overline{OR}$ (sd)	$\overline{FP}$ (sd)	$\overline{dFP}$ (sd)	$\overline{FPC}$ (sd)	Distribution DE genes according with Δ_g
50	8.7	C_1^*	17.7 (7.6)	146.6 (35.2)	0.0 (0.1)	0.0 (0.0)	0.0 (0.3)	(3.5, 17.7, 78.8)
		C_2^*	29.9 (6.0)	45.8 (10.6)	1.5 (1.5)	1.1 (1.8)	2.6 (8.9)	(47.6, 42.7, 9.6)
		C_3	27.7 (4.8)	9.2 (0.8)	9.2 (0.2)	23.4 (2.3)	89.3 (6.4)	(94.5, 5.5, 0)
100	8.1	C_1^*	31.8 (11.1)	50.3 (6.6)	0.0 (0.0)	0.0 (0.0)	0.0 (0.0)	(1.9, 15.6, 82.4)
		C_2^*	59.6 (10.8)	23.5 (2.7)	1.1 (0.7)	0.4 (0.2)	0.3 (0.8)	(44.2, 43.8, 12.0)
		C_3	24.6 (9.2)	9.1 (0.7)	8.7 (0.4)	19.5 (2.1)	76.4 (9.8)	(95.9, 4.1, 0)
200	7.1	C_1^*	32.5 (14.1)	33.5 (3.3)	0.0 (0.0)	0.0 (0.0)	0.0 (0)	(1.8, 14.8, 83.4)
		C_2^*	116.8(13.4)	16.1 (1.2)	0.4 (0.1)	0.2 (0.1)	0.1 (0.3)	(43.5, 44.5, 12.0)
		C_3	21.1 (6.3)	8.1 (0.6)	6.2 (1.1)	9.3 (2.2)	25.1 (13.2)	(95.4, 4.6, 0)

Table 20 Simulation study 1, scenario 1 with $n_X = 30$, $n_Y = 10$ and 100 runs. Evaluation of the second step of the ORdensity method with $\alpha = 0.05$. In the first two columns, the delta Δ values and number of total simulated DE genes. In column 3, the $10 \times f_0$ values where f_0 is the average proportion of permuted cases in sets U_i. In column 4, the "*" indicates the clusters considered by the procedure. Columns 5-8 contain for each cluster: the mean number of genes ($n^-{}_i$), the mean of OR values $\overline{(OR)}$, the mean of FP values $\overline{(FP)}$, the mean of dFP values ($\overline{dFP}$). In the last column the mean of False Positive genes per cluster in % ($\overline{FPC}$). Corresponding standard deviations are in brackets.

Δ	DE	$10f_0$	C_i	$\bar{n}_i$ (sd)	$\overline{OR}$ (sd)	$\overline{FP}$ (sd)	$\overline{dFP}$ (sd)	$\overline{FPC}$ (sd)
$\Delta = 1.5$	50	8.6	C_1^*	21.9 (5.5)	33.6 (5.7)	3.5 (0.8)	2.5 (1.0)	4.5 (4.6)
			C_2^*	29.7 (6.9)	14.2 (2.1)	7.7 (0.7)	11.4 (2.3)	35.3 (12.3)
			C_3	28.3 (7.5)	8.6 (0.5)	9.3 (0.2)	23.9 (2.2)	80.9 (8.6)
	100	8.6	C_1^*	35.1 (8.7)	30.1 (3.8)	1.7 (0.4)	1.4 (0.5)	1.4 (2.1)
			C_2^*	38.1 (6.5)	14.3 (1.7)	5.5 (0.9)	7.3 (1.7)	10.6 (5.6)
			C_3	35.7 (7.9)	8.9 (0.5)	8.6 (0.4)	18.4 (2.0)	48.6 (10.1)
	200	7.7	C_1^*	47.9 (12.1)	22.9 (2.4)	0.6 (0.2)	0.5 (0.2)	0.2 (0.7)
			C_2^*	60.3 (11.6)	11.8 (0.9)	2.4 (0.6)	3.2 (0.8)	2.1 (1.9)
			C_3	38.9 (8.3)	8.2 (0.3)	6.1 (0.6)	10.1 (1.2)	10.2 (5.2)
$\Delta = 2$	50	8.5	C_1^*	29.6 (5.9)	47.2 (7.6)	1.2 (0.8)	0.8 (0.4)	0.7 (1.4)
			C_2^*	24.0 (4.5)	18.9 (4.3)	6.5 (1.6)	7.4 (2.9)	27.0 (16.0)
			C_3	31.6 (6.8)	9.0 (0.7)	9.2 (0.2)	22.5 (2.4)	91.4 (6.1)
	100	8.1	C_1^*	40.6 (13.4)	46.6 (7.9)	0.2 (0.1)	0.2 (0.1)	0.1 (0.5)
			C_2^*	44.2 (12.0)	21.2 (3.3)	2.8 (1.2)	2.8 (1.3)	3.8 (3.2)
			C_3	35.4 (6.2)	10.0 (0.7)	8.6 (0.4)	15.9 (1.9)	57.9 (10.1)
	200	7.3	C_1^*	53.6 (18.2)	32.3 (4.4)	0.1 (0.0)	0.1 (0.0)	0.1 (0.3)
			C_2^*	99.3 (16.0)	15.3 (1.6)	0.8 (0.3)	1.0 (0.4)	0.5 (0.8)
			C_3	35.6 (8.7)	8.6 (0.5)	5.5 (0.8)	7.3 (1.2)	10.4 (5.2)
$\Delta = 3$	50	8.5	C_1^*	23.5 (11.1)	114.4 (25.6)	0.1 (0.1)	0.0 (0.1)	0.0 (0.0)
			C_2^*	27.6 (7.9)	48.7 (14.8)	2.1 (2.5)	1.9 (3.6)	9.9 (24.5)
			C_3	36.6 (6.1)	10.0 (0.9)	9.2 (0.2)	21.3 (2.1)	97.0(3.0)
	100	8.0	C_1^*	33.3 (5.7)	100.7 (14.0)	0.0 (0.0)	0.0 (0.0)	0.0 (0.0)
			C_2^*	64.5 (8.7)	45.9 (4.8)	0.5 (0.2)	0.3 (0.1)	0.5 (1.1)
			C_3	27.0 (5.1)	10.0 (0.7)	9.1 (0.3)	18.0 (1.5)	90.9 (5.8)
	200	7.1	C_1^*	65.8 (12.2)	59.0 (6.2)	0.0 (0.0)	0.0 (0.0)	0.0 (0.0)
			C_2^*	126.0 (15.7)	27.6 (2.8)	0.3 (0.1)	0.2 (0.1)	0.2 (0.4)
			C_3	13.6 (14.6)	10.0 (2.5)	7.7 (2.0)	8.9 (3.1)	53.7 (26.2)

Table 21 Simulation study 1, scenario 2 with $n_X = 30$, $n_Y = 10$ and 100 runs. Evaluation of the second step of the ORdensity method with $\alpha = 0.05$. In the first two columns, the delta Δ values and number of total simulated DE genes. In column 3, the $10 \times f_0$ values where f_0 is the average proportion of permuted cases in sets U_i. In column 4, the "*" indicates the clusters considered by the procedure. Columns 5-8 contain for each cluster: the mean number of genes ($n^-{}_i$), the mean of OR values $(\overline{OR})$, the mean of FP values $(\overline{FP})$, the mean of dFP values $(\overline{dFP})$. In the last column the mean of False Positive genes per cluster in % $(\overline{FPC})$. Corresponding standard deviations are in brackets.

Δ	DE	$10f_0$	C_i	$\bar{n}_i$ (sd)	$\overline{OR}$ (sd)	$\overline{FP}$ (sd)	$\overline{dFP}$ (sd)	$\overline{FPC}$ (sd)
			C_1^*	23.4 (5.8)	38.4 (7.4)	2.6 (0.8)	1.7 (0.8)	3.8 (4.1)
	50	8.6	C_2^*	28.7 (5.4)	15.1 (2.3)	7.1 (0.8)	8.9 (2.1)	32.3 (12.9)
			C_3	28.1 (7.4)	8.8 (0.5)	9.2 (0.2)	21.6 (2.1)	81.5 (8.2)
			C_1^*	37.0 (8.7)	35.3 (4.8)	1.0 (0.4)	0.7 (0.4)	1.7 (2.2)
1.5	100	8.1	C_2^*	39.2 (6.1)	15.5 (2.3)	4.6 (1.0)	5.3 (1.6)	9.6 (5.9)
			C_3	36.3 (6.6)	8.9 (0.5)	8.5 (0.4)	16.0 (2.0)	48.1 (8.9)
			C_1^*	53.0 (16.2)	26.5 (4.3)	0.2 (0.1)	0.3 (0.1)	0.5 (1.0)
	200	7.6	C_2^*	68.5 (12.7)	12.3 (1.3)	1.8 (0.5)	2.2 (0.7)	2.3 (2.0)
			C_3	39.6 (9.4)	8.2 (0.4)	5.8 (0.6)	8.4 (1.1)	12.3 (5.4)
			C_1^*	30.3 (7.0)	54.1 (9.1)	0.8 (0.4)	0.4 (0.3)	1.2 (2.2)
	50	8.6	C_2^*	24.4 (4.6)	19.9 (5.0)	5.9 (1.8)	6.1 (2.8)	28.0 (18.0)
			C_3	30.0 (6.0)	9.1 (0.8)	9.2 (0.2)	20.8 (2.3)	92.8 (5.8)
			C_1^*	37.8 (14.5)	57.7 (10.7)	0.1 (0.1)	0.1 (0.1)	0.1 (0.7)
2	100	8.0	C_2^*	51.7 (12.0)	24.0 (4.2)	2.2 (1.2)	1.9 (1.1)	4.2 (3.8)
			C_3	32.6 (5.6)	10.1 (0.8)	8.6 (0.4)	14.7 (1.6)	64.9 (9.8)
			C_1^*	55.3 (19.8)	40.6 (6.2)	0.0 (0.1)	0.0 (0.0)	0.1 (0.3)
	200	7.2	C_2^*	110.9 (17.8)	17.0 (1.8)	0.7 (0.4)	0.7 (0.4)	0.9 (1.0)
			C_3	30.3 (7.5)	8.7 (0.5)	5.8 (0.9)	7.0 (1.3)	16.3 (8.5)
			C_1^*	22.6 (12.3)	144.8 (42.6)	0.0 (0.1)	0.0 (0.0)	0.2 (0.7)
	50	8.5	C_2^*	29.3 (7.8)	53.9 (18.6)	2.0 (2.7)	1.8 (3.6)	13.6 (28.3)
			C_3	34.7 (6.1)	10.2 (1.1)	9.2 (0.2)	19.7 (2.2)	96.8 (3.2)
			C_1^*	31.2 (9.8)	132.4 (24.7)	0.0 (0.0)	0.0 (0.0)	0.0 (0.0)
3	100	8.0	C_2^*	67.6 (9.8)	53.0 (5.6)	0.4 (0.2)	0.2 (0.1)	0.9 (1.3)
			C_3	27.5 (4.8)	10.1 (0.8)	9.1 (0.3)	16.9 (1.3)	93.6 (4.8)
			C_1^*	59.9 (12.7)	79.0 (8.8)	0.0 (0.0)	0.0 (0.0)	0.0 (0.1)
	200	7.1	C_2^*	136.9 (14.4)	31.9 (2.7)	0.2 (0.1)	0.1 (0.1)	0.4 (0.6)
			C_3	10.8 (9.5)	9.0 (1.8)	8.6 (1.1)	10.0 (2.1)	72.8 (17.5)

Table 22 Simulation study 1, scenario 3 with $n_X = 30$, $n_Y = 10$ and 100 runs. Evaluation of the second step of the ORdensity method with $\alpha = 0.05$. In the first column the number of total simulated DE genes. In column 2, the $10 \times f_0$ values where f_0 is the average proportion of permuted cases in sets U_i. In column 3, the "*" indicates the clusters considered by the procedure. Columns 4-7 contain for each cluster: the mean number of genes ($n^-{}_i$), the mean of OR values $(\overline{OR})$, the mean of FP values $(\overline{FP})$, the mean of dFP values (dFP). In column 8 the mean of False Positive genes per cluster in % $(\overline{FPC})$. Corresponding standard deviations are in brackets. In the last column, distribution of DE genes in accordance with their Δ_g value that they have taken by chance in $\{1.5, 2, 3\}$.

DE	$10f_0$	C_i	$\bar{n}_i$ (sd)	$\overline{OR}$ (sd)	$\overline{FP}$ (sd)	$\overline{dFP}$ (sd)	$\overline{FPC}$ (sd)	Distribution DE genes according to Δ_g
		C_1^*	29.8 (5.4)	77.0 (13.7)	0.7 (0.4)	0.3 (0.2)	1.1 (2.0)	(13.3, 31.2, 55.5)
50	8.6	C_2^*	25.0 (5.1)	19.6 (5.1)	6.5 (1.4)	6.7 (2.5)	32.8 (15.6)	(55.2, 39.2, 5.6)
		C_3	29.3 (6.6)	9.0 (0.7)	9.3 (0.2)	21.3 (2.1)	89.3 (7.6)	(85.6, 14.3, 0.1)
		C_1^*	39.3 (14.4)	80.7 (16.9)	0.1 (0.1)	0.1 (0.1)	1.1 (2.0)	(7.8, 23.2, 69.1)
100	8.1	C_2^*	44.7 (11.7)	16.2 (6.6)	2.5 (1.5)	2.3 (1.6)	5.7 (5.9)	(39.9, 45.1, 15.0)
		C_3	36.8 (6.5)	10.0 (0.8)	8.5 (0.4)	15.0 (1.7)	60.0 (9.3)	(75.3, 24.4, 0.3)
		C_1^*	46.2 (17.3)	65.1 (13.0)	0.0 (0.0)	0.0 (0.0)	0.0 (0.2)	(3.7, 14.8, 81.5)
200	7.3	C_2^*	107.0 (17.4)	21.2 (2.9)	0.7 (0.3)	0.6 (0.4)	1.0 (1.1)	(28.8, 43.8, 27.4)
		C_3	35.5 (7.5)	8.8 (0.5)	6.0 (0.7)	7.5 (1.1)	16.6 (6.6)	(67.9, 30.8, 1.3)

Table 23 Simulation study 1, scenario 3 with $n_X = n_Y = 10$ and 100 runs. Evaluation of the second step of the ORdensity method with $\alpha = 0.05$. In the first column the number of total simulated DE genes. In column 2, the $10 \times f_0$ values where f_0 is the average proportion of permuted cases in sets U_i. In column 3, the "*" indicates the clusters considered by the procedure. Columns 4-7 contain for each cluster: the mean number of genes $(\bar{n}_i)$, the mean of OR values $(\overline{OR})$, the mean of FP values $(\overline{FP})$, the mean of dFP values $(\overline{dFP})$. In column 8 the mean of False Positive genes per cluster in % $(\overline{FPC})$. Corresponding standard deviations are in brackets. In the last column, distribution of DE genes in accordance with their Δ_g value that they have taken by chance in $\{1.5, 2, 3\}$.

DE	$10f_0$	C_i	$\bar{n}_i$ (sd)	$\overline{OR}$ (sd)	$\overline{FP}$ (sd)	$\overline{dFP}$ (sd)	$\overline{FPC}$ (sd)	Distribution DE genes according to Δ_g
		C_1^*	21.6 (5.3)	77.7 (22.2)	2.8 (0.7)	1.0 (0.5)	3.4 (4.2)	(11.2, 27.5, 61.2)
50	8.6	C_2^*	30.0 (5.5)	20.5 (5.5)	7.5 (0.9)	7.5 (1.9)	34.4 (12.8)	(37.2, 42.3, 20.5)
		C_3	29.8 (8.4)	10.0 (0.8)	9.2 (0.3)	17.8 (1.7)	83.0 (8.2)	(63.7, 34.6, 1.7)
		C_1^*	38.3 (8.7)	67.1 (12.0)	1.4 (0.5)	0.6 (0.3)	1.1 (1.7)	(10.4, 25.8, 63.8)
100	8.2	C_2^*	36.6 (6.1)	20.9 (4.1)	5.4 (1.1)	4.8 (1.5)	11.9 (7.1)	(31.1, 43.0, 25.8)
		C_3	35.6 (8.2)	10.4 (0.9)	8.8 (0.3)	13.8 (1.5)	52.2 (11.1)	(59.0, 36.3, 4.7)
		C_1^*	55.8 (17.0)	51.4 (12.6)	0.6 (0.3)	0.3 (0.2)	0.3 (0.6)	(8.2, 22.5, 69.3)
200	7.6	C_2^*	60.6 (14.3)	18.0 (3.5)	2.6 (0.7)	2.2 (0.7)	2.1 (2.1)	(23.0, 38.2, 38.8)
		C_3	42.6 (9.6)	9.8 (0.7)	6.6 (0.5)	7.6 (0.9)	14.4 (5.4)	(44.1, 43.8, 12.1)

Table 24 AUC mean values for Simulation study 1, scenario 2 and 100 runs

$n_X = n_Y = 30$

Nb. of DE genes		50			100			200	
Δ	1.5	2	3	1.5	2	3	1.5	2	3
ORdensity	0.998	0.997	0.997	0.998	0.997	0.995	0.998	0.996	0.993
	(0.001)	(0.001)	(0.000)	(0.001)	(0.002)	(0.000)	(0.001)	(0.003)	(0.000)
limma	0.995	0.993	0.993	0.996	0.993	0.993	0.997	0.992	0.992
	(0.002)	(0.001)	(0.000)	(0.003)	(0.001)	(0.000)	(0.003)	(0.001)	(0.000)
SAM	0.995	0.994	0.993	0.996	0.993	0.993	0.997	0.993	0.992
	(0.002)	(0.001)	(0.000)	(0.003)	(0.001)	(0.000)	(0.003)	(0.002)	(0.000)

$n_X = 30,\ n_Y = 10$

Nb. of DE genes		50			100			200	
Δ	1.5	2	3	1.5	2	3	1.5	2	3
ORdensity	0.981	0.997	0.998	0.980	0.997	0.998	0.971	0.995	0.998
	(0.008)	(0.003)	(0.001)	(0.005)	(0.002)	(0.002)	(0.006)	(0.001)	(0.003)
limma	0.976	0.997	0.993	0.987	0.998	0.993	0.995	0.999	0.992
	(0.010)	(0.003)	(0.001)	(0.005)	(0.002)	(0.001)	(0.001)	(0.001)	(0.001)
SAM	0.949	0.993	0.993	0.956	0.994	0.993	0.970	0.997	0.992
	(0.015)	(0.005)	(0.001)	(0.011)	(0.003)	(0.001)	(0.008)	(0.002)	(0.001)

$n_X = n_Y = 10$

Nb. of DE genes		50			100			200	
Δ	1.5	2	3	1.5	2	3	1.5	2	3
ORdensity	0.943	0.983	0.998	0.939	0.981	0.998	0.921	0.972	0.998
	(0.013)	(0.007)	(0.001)	(0.010)	(0.006)	(0.001)	(0.010)	(0.006)	(0.001)
limma	0.912	0.984	0.994	0.944	0.991	0.994	0.976	0.997	0.995
	(0.021)	(0.008)	(0.002)	(0.011)	(0.004)	(0.002)	(0.005)	(0.001)	(0.004)
SAM	0.885	0.967	0.994	0.890	0.968	0.994	0.02	0.981	0.996
	(0.020)	(0.016)	(0.002)	(0.015)	(0.009)	(0.002)	(0.014)	(0.005)	(0.004)

Table 25 AUC mean values for Simulation study 1, scenario 3 and 100 runs

$n_X = n_Y = 30$

Nb. of DE genes	50	100	200
		Δ variable	
ORdensity	0.998	0.998	0.999
	(0.001)	(0.002)	(0.001)
limma	0.994	0.995	0.995
	(0.002)	(0.003)	(0.003)
SAM	0.994	0.995	0.996
	(0.002)	(0.003)	(0.003)

$n_X = 30,\ n_Y = 10$

Nb. of DE genes	50	100	200
		Δ variable	
ORdensity	0.993	0.992	0.991
	(0.004)	(0.003)	(0.002)
limma	0.991	0.994	0.997
	(0.005)	(0.003)	(0.001)
SAM	0.980	0.983	0.989
	(0.010)	(0.006)	(0.004)

$n_X = n_Y = 10$

Nb. of DE genes	50	100	200
		Δ variable	
ORdensity	0.975	0.974	0.968
	(0.009)	(0.006)	(0.005)
limma	0.967	0.980	0.993
	(0.012)	(0.006)	(0.002)
SAM	0.950	0.956	0.971
	(0.015)	(0.009)	(0.006)

Table 26 Simulation study 2 with $n_X = 30,\ n_Y = 10$ and 100 runs. Evaluation of the first step of the ORdensity method using different values of α. The Table shows the estimated probability, $\hat{p}_m$, of no considering as a potential DE gene at least one gene that it really is, and the mean proportion of DE genes (row named "%") that the procedure considered as potential DE genes.

Nb. of DE genes per block		1 block			2 blocks			3 blocks		
			α			α			α	
		0.1	0.05	0.01	0.1	0.05	0.01	0.1	0.05	0.01
5	$\hat{p}_m$	0	0	0	0.01	0.01	0.04	0.04	0.06	0.29
	%	100 (0)	100 (0)	100 (0)	99.9 (1.0)	99.9 (1.0)	99.2 (4.6)	99.6 (2.3)	99.1 (3.9)	95.2 (10.0)
20	$\hat{p}_m$	0	0	0	0.00	0.00	0.07	0.08	0.15	0.53
	%	100 (0)	100 (0)	100 (0)	100 (0)	100 (0)	99.6 (2.3)	99.6 (1.8)	98.8 (4.2)	93.7 (10.3)
100	$\hat{p}_m$	0	0	0	0.02	0.06	0.30	0.17	0.42	0.91
	%	100 (0)	100 (0)	100 (0)	99.9 (0.1)	99.7 (1.6)	97.3 (8.2)	99.5 (1.6)	98.0 (5.1)	87.5 (14.3)

Table 27 Simulation study 2, with $n_X = 30$, $n_Y = 10$ and 100 runs. Evaluation of the second step of the ORdensity method with $\alpha = 0.05$. In the first column number of blocks. In column 2, the number of total simulated DE genes. In column 3, the $10 \times f_0$ values where f_0 is the average proportion of permuted cases in sets U_i. In column 4, the "*" indicates the clusters considered by the procedure. Columns 5-8 contain for each cluster: the mean number of genes ($n^-{}_i$), the mean of OR values $\overline{(OR)}$, the mean of FP values $\overline{(FP)}$, the mean of dFP values $\overline{(dFP)}$. In the last column the mean of False Positive genes per cluster in % ($\overline{FPC}$). Corresponding standard deviations are in brackets.

B	DE	$10f_0$	C_i	$\bar{n}_i$ (sd)	$\overline{OR}$ (sd)	$\overline{FP}$ (sd)	$\overline{dFP}$ (sd)	$\overline{FPC}$ (sd)
1	5	9.1	C_1^*	29.7 (32.1)	94.8 (61.2)	6.4 (1.2)	6.7 (8.7)	33.0 (44.3)
			C_2	88.1 (26.1)	10.7 (0.7)	9.1 (0.3)	18.2 (2.8)	100.0 (0.0)
			C_3	85.8 (19.0)	8.1 (0.3)	9.4 (0.3)	32.7 (1.8)	100.0 (0.0)
	20	9.0	C_1^*	20.0 (0.0)	127.9 (31.3)	0.0 (0.0)	0.0 (0.0)	0 (0)
			C_2	100.7 (13.7)	11.0 (0.5)	9.0 (0.1)	16.9 (1.5)	100 (0)
			C_3	85.8 (19.0)	8.1 (0.3)	9.2 (0.1)	32.7 (1.8)	100 (0)
	100	8.9	C_1^*	100.0 (0.0)	120.0 (26.5)	0.0 (0.0)	0.0 (0.0)	0 (0)
			C_2	84.5 (12.4)	11.0 (0.5)	9.1 (0.2)	16.5 (1.2)	100(0)
			C_3	77.0 (10.6)	8.1 (0.2)	9.2 (0.1)	30.3 (1.3)	100 (0)
2	10	9.1	C_1^*	10.4 (8.5)	72.3 (19.9)	1.6 (1.2)	0.7 (2.3)	2.6 (0.1)
			C_2	103.8 (14.5)	11.2 (0.7)	9.0 (0.2)	16.7 (1.5)	99.9 (1.6)
			C_3	95.0 (14.6)	8.0 (0.2)	9.2 (0.1)	32.3 (1.2)	100.0 (0.0)
	40	8.9	C_1^*	39.7 (1.3)	65.5 (11.9)	0.1 (0.2)	0.1 (0.2)	0.1 (0.4)
			C_2	98.9 (12.0)	11.0 (0.5)	9.0 (0.2)	17.0 (1.2)	99.6 (1.2)
			C_3	88.0 (11.9)	8.0 (0.2)	9.2 (0.1)	32.3 (1.2)	100.0 (0.0)
	200	8.6	C_1^*	100.7 (21.8)	68.7 (17.6)	0.0 (0.0)	0.0 (0.0)	0.0 (0.0)
			C_2^*	102.5 (8.9)	42.5 (13.6)	0.6 (1.8)	1.0 (3.4)	4.2 (19.7)
			C_3	141.5 (19.2)	9.3 (0.4)	9.2 (0.1)	23.9 (1.6)	99.7 (0.9)
3	15	9.1	C_1^*	13.5 (5.4)	52.0 (10.8)	1.4 (1.2)	0.7 (1.2)	1.9 (8.8)
			C_2	101.4 (14.6)	11.2 (0.6)	9.0(0.1)	16.9 (1.3)	97.9 (2.2)
			C_3	94.8 (13.8)	8.0 (0.2)	9.2 (0.2)	32.5 (1.1)	100.0 (0.1)
	60	8.9	C_1^*	55.4 (6.3)	47.8 (9.2)	0.5 (0.4)	0.4 (0.4)	0.3 (0.9)
			C_2	97.5 (13.8)	11.2 (0.7)	9.0 (0.2)	16.8 (1.3)	95.4 (5.7)
			C_3	87.4 (12.7)	7.8 (0.2)	9.2 (0.1)	32.3 (1.1)	99.8 (0.9)
	300	8.3	C_1^*	138.6 (31.9)	47.2 (11.9)	0.0 (0.1)	0.0 (0.1)	0.0 (0.0)
			C_2^*	156.8 (32.8)	27.3 (7.3)	0.8 (1.0)	1.2 (1.8)	1.2 (4.2)
			C_3	130.4 (12.8)	9.1 (0.3)	9.2 (0.2)	23.2 (1.0)	95.6 (6.0)

FIGURES 8 – 20

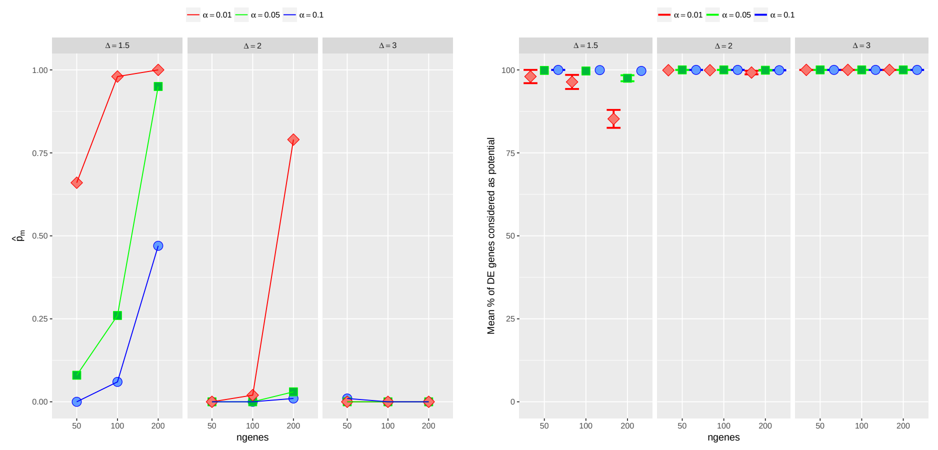

Figure 8 Simulation study 1, scenario 2 with $n_X = n_Y = 30$ and 100 runs. Evaluation of the first step of the ORdensity method using different values of α. Left: estimated probability, $\hat{p}_m$, of no considering as *potential* DE gene at least one gene that it really is. Right: mean proportion of DE genes that the procedure considered as *potential* DE genes.

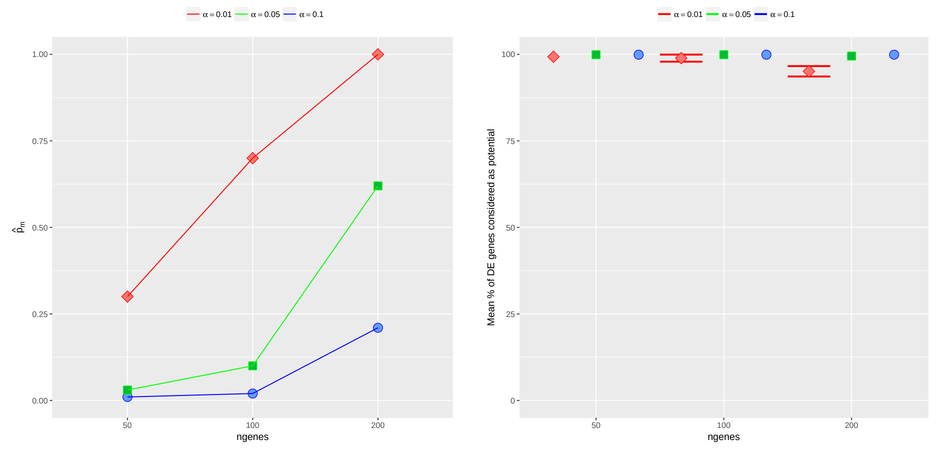

Figure 9 Simulation study 1, scenario 3 with $n_X = n_Y = 30$ and 100 runs. Evaluation of the first step of the ORdensity method using different values of α. Left: estimated probability, $\hat{p}_m$, of no considering as *potential* DE gene at least one gene that it really is. Right: mean proportion of DE genes that the procedure considered as *potential* DE genes.

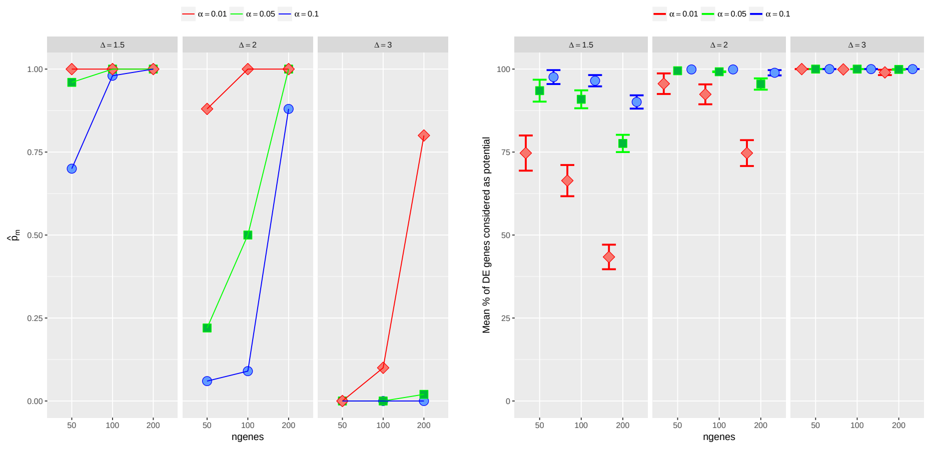

Figure 10 Simulation study 1, scenario 2 with $n_X = 30$, $n_Y = 10$ and 100 runs. Evaluation of the first step of the ORdensity method using different values of α. Left: estimated probability, $\hat{p}_m$, of no considering as *potential* DE gene at least one gene that it really is. Right: mean proportion of DE genes that the procedure considered as *potential* DE genes.

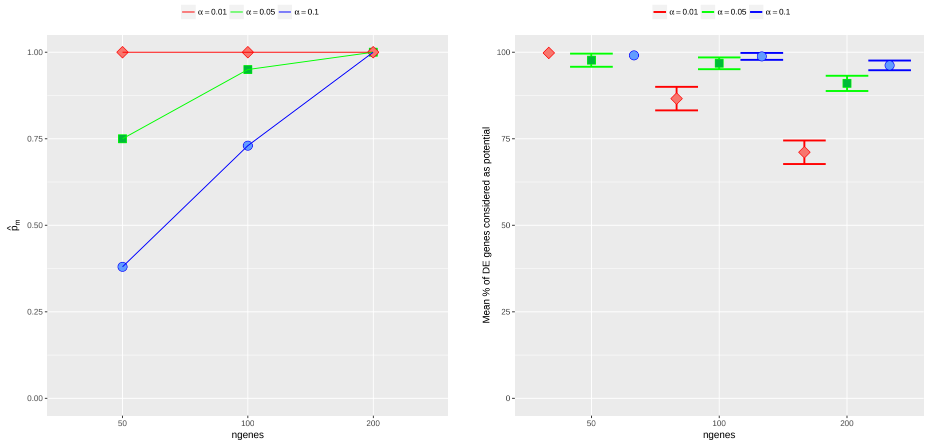

Figure 11 Simulation study 1, scenario 3 with $n_X = 30$, $n_Y = 10$ and 100 runs. Evaluation of the first step of the ORdensity method using different values of α. Left: estimated probability, $\hat{p}_m$, of no considering as *potential* DE gene at least one gene that it really is. Right: mean proportion of DE genes that the procedure considered as *potential* DE genes.

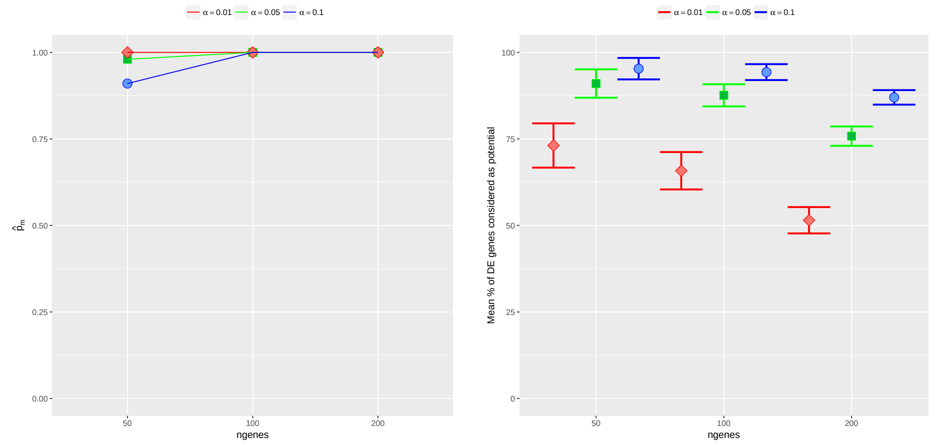

Figure 12 Simulation study 1, scenario 3 with $n_X = 10$, $n_Y = 10$ and 100 runs. Evaluation of the first step of the ORdensity method using different values of α. Left: estimated probability, $\hat{p}_m$, of no considering as *potential* DE gene at least one gene that it really is. Right: mean proportion of DE genes that the procedure considered as *potential* DE genes.

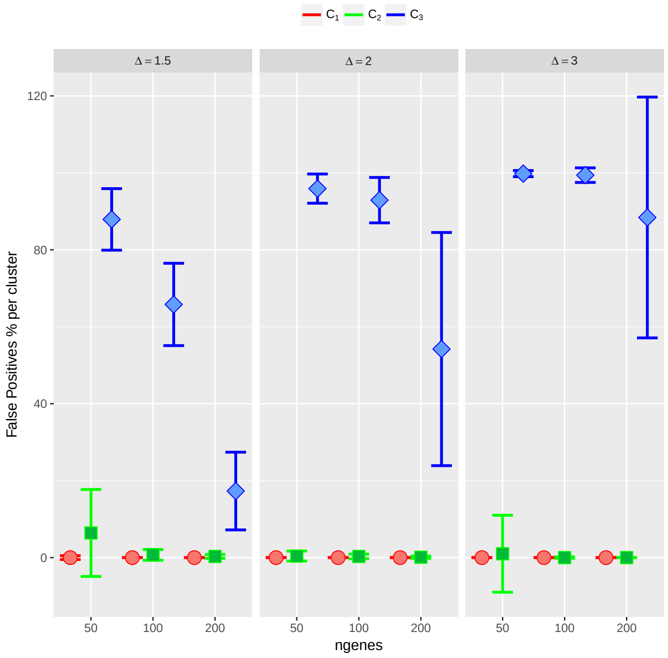

Figure 13 Simulation study 1, scenario 2 with $n_X = n_Y = 30$ and 100 runs. Evaluation of the second step of the ORdensity method with $\alpha = 0.05$. In x axis number of DE genes; in y axis the mean of False Positive genes per cluster in % ($\overline{FPC}$).

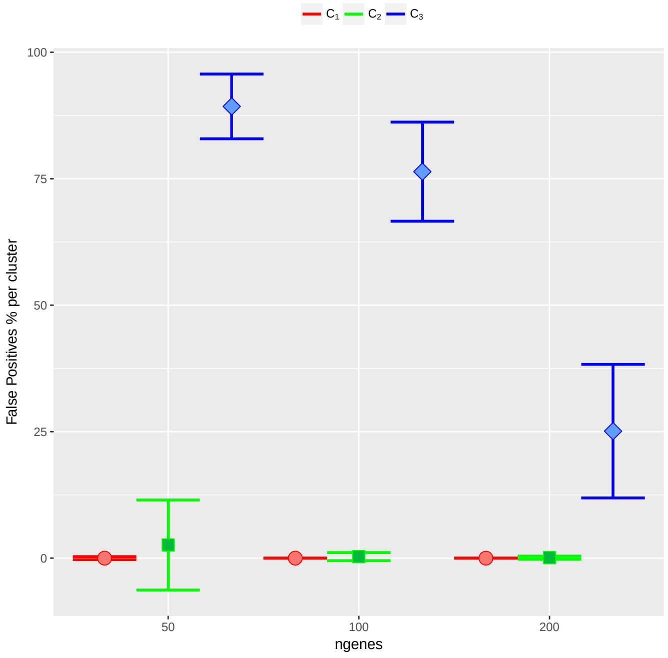

Figure 14 Simulation study 1, scenario 3 with $n_X = n_Y = 30$ and 100 runs. Evaluation of the second step of the ORdensity method with $\alpha = 0.05$. In x axis number of DE genes; in y axis the mean of False Positive genes per cluster in % ($\overline{FPC}$).

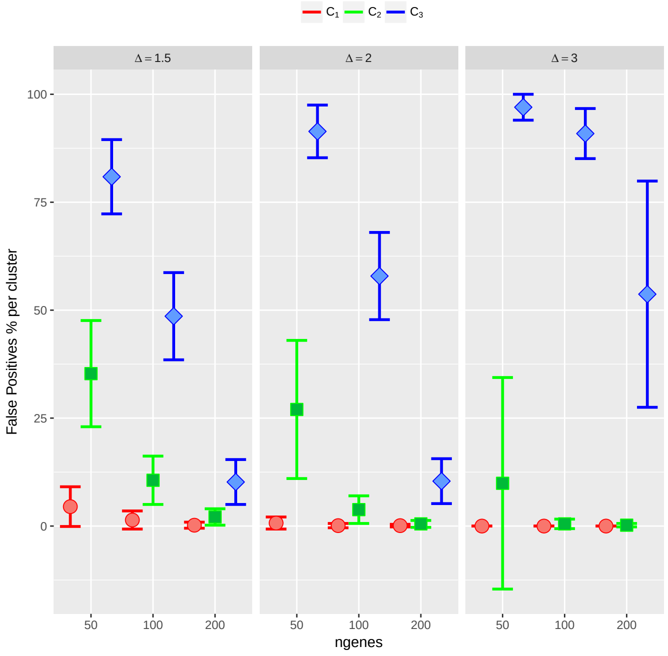

Figure 15 Simulation study 1, scenario 1 with $n_X = 30$, $n_Y = 10$ and 100 runs. Evaluation of the second step of the ORdensity method with $\alpha = 0.05$.In x axis number of DE genes; in y axis the mean of False Positive genes per cluster in % ($\overline{FPC}$).

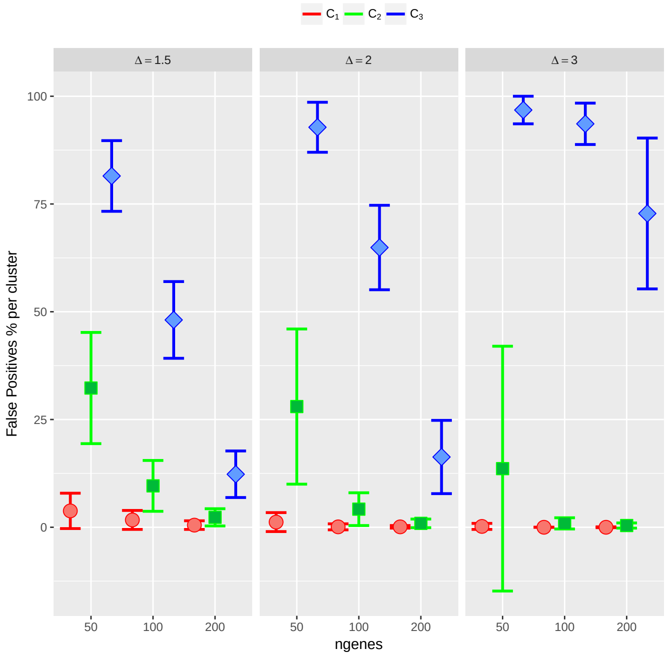

Figure 16 Simulation study 1, scenario 2 with $n_X = 30$, $n_Y = 10$ and 100 runs. Evaluation of the second step of the ORdensity method with $\alpha = 0.05$. In x axis number of DE genes; in y axis the mean of False Positive genes per cluster in % ($\overline{FPC}$).

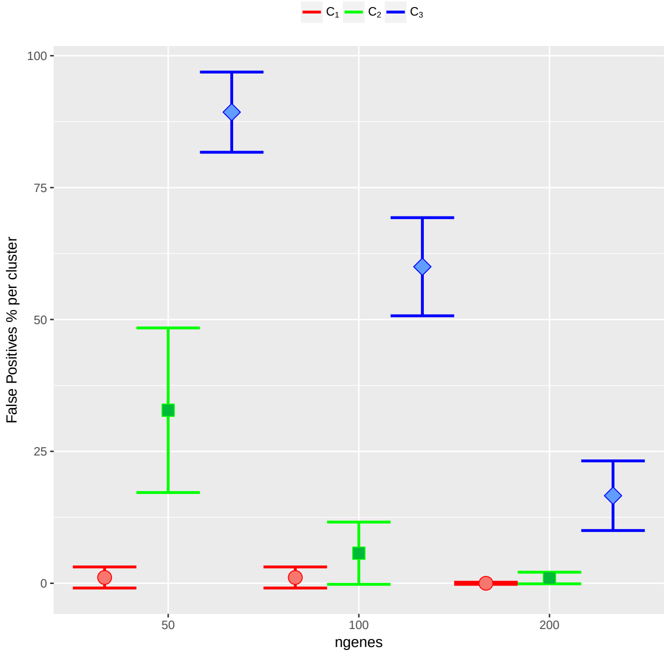

Figure 17 Simulation study 1, scenario 3 with $n_X = 30$, $n_Y = 10$ and 100 runs. Evaluation of the second step of the ORdensity method with $\alpha = 0.05$. In x axis number of DE genes; in y axis the mean of False Positive genes per cluster in % ($\overline{FPC}$).

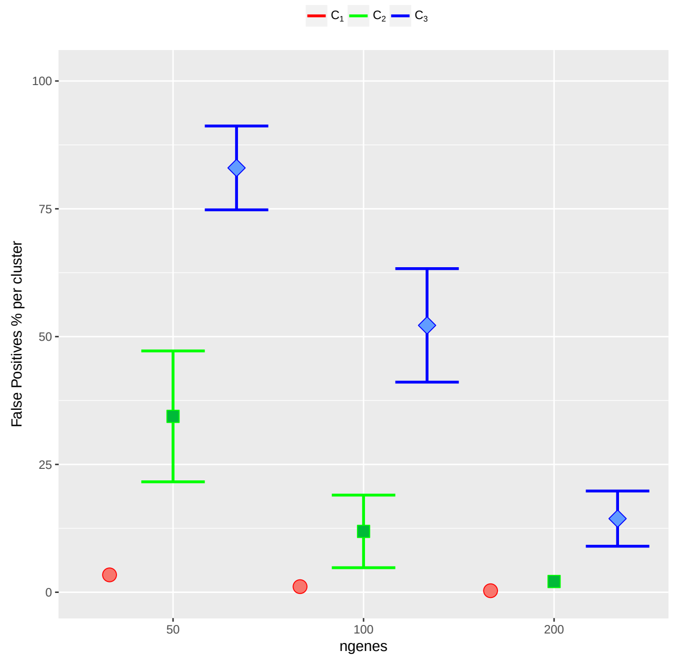

Figure 18 Simulation study 1, scenario 3 with $n_X = n_Y = 10$ and 100 runs. Evaluation of the second step of the ORdensity method with $\alpha = 0.05$. In x axis number of DE genes; in y axis the mean of False Positive genes per cluster in % $(\overline{FPC})$.

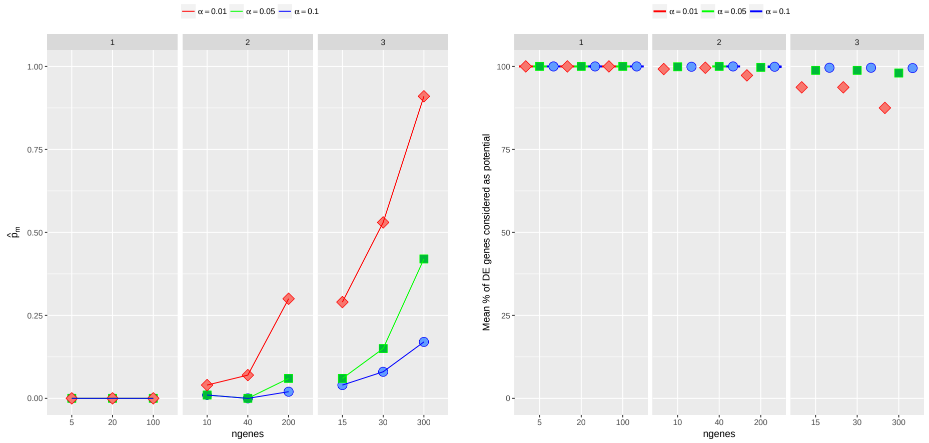

Figure 19 Simulation study 2, with $n_X = 30$, $n_Y = 10$ and 100 runs. Evaluation of the first step of the ORdensity method using different values of α. Left: estimated probability, $\hat{p}_m$, of no considering as *potential* DE gene at least one gene that it really is. Right: mean proportion of DE genes that the procedure considered as *potential* DE genes.

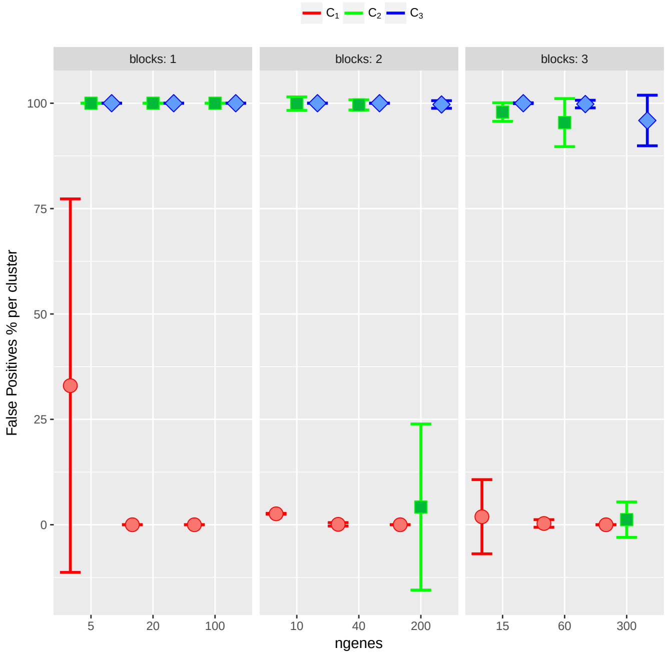

Figure 20 Simulation study 2 with $n_X = 30$, $n_Y = 10$ and 100 runs. Evaluation of the second step of the ORdensity method with $\alpha = 0.05$. In x axis number of DE gens; in y axis the mean of False Positive genes per cluster in % ($\overline{FPC}$).
